# Isolation of the Elusive Heptavanadate Anion with Trisalkoxide Ligands

**DOI:** 10.1021/acs.inorgchem.1c00448

**Published:** 2021-04-05

**Authors:** Leticia Fernández-Navarro, Aitor Nunes-Collado, Beñat Artetxe, Estibaliz Ruiz-Bilbao, Leire San Felices, Santiago Reinoso, Ana San José Wéry, Juan M. Gutiérrez-Zorrilla

**Affiliations:** ^†^Departamento de Química Inorgánica, and ^‡^Servicios Generales de Investigación SGIker, Facultad de Ciencia y Tecnología, Universidad del País Vasco UPV/EHU, P.O. Box 644, 48080 Bilbao, Spain; § Departamento de Ciencias and Institute for Advanced Materials and Mathematics (InaMat^2^), Universidad Pública de Navarra (UPNA), Campus de Arrosadia, 31006 Pamplona, Spain; ∥ Departamento de Desarrollo Sostenible, Universidad Católica de Ávila, c/Canteros s/n, 05005 Ávila, Spain; ⊥ BCMaterials, Basque Center for Materials, Applications and Nanostructures, UPV/EHU Science Park, 48940 Leioa, Spain

## Abstract

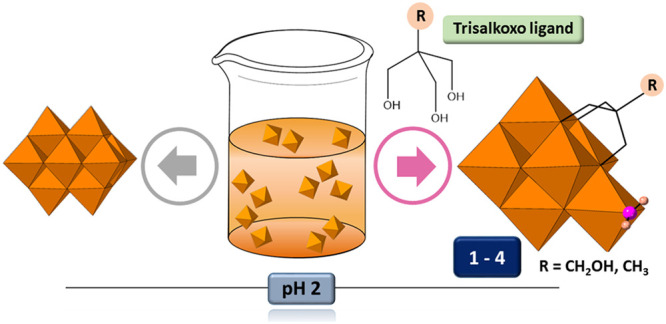

The unprecedented heptavanadate cluster has been isolated from reactions between trisalkoxide ligands and vanadate in water at pH = 2 as a series of alkylammonium [H_*x*_V_7_O_18_(H_2_O)((OCH_2_)_3_CR)]^(4–x)-^ salts (**1**–**3**, R = CH_2_OH; **4**, R = CH_3_). Their structures have been determined and the partial stability of **4** in water assessed by a combination of multinuclear NMR spectroscopy and ESI-MS. The heptavanadate unit reported herein could represent an intermediate species in the formation of decavanadate that is blocked by attachment of tripodal ligands.

## Abstract 2

The elusive heptavanadate anion has been isolated from the reaction between trisalkoxide ligands and a vanadate source in acidic aqueous solution. A series of alkylammonium salts of the monofunctionalized [H_*x*_V_7_O_18_(H_2_O)((OCH_2_)_3_CR)]^(4−x)−^ anion (**1**−**3**, R = CH_2_OH; **4**, R = CH_3_) has been structurally characterized. The partial stability of the hybrid anion in water has been addressed by a combination of multinuclear NMR spectroscopy and ESI-MS, which reveals that the nude heptavanadate core rapidly rearranges into the well-known decavanadate anion.

Organic functionalization of polyoxometalates (POMs) is the key for these anionic metal-oxo clusters to be covalently immobilized into matrices like polymers or carbons and also to interact with diverse surfaces (i.e., metals and their oxides).^1^ Different routes have been investigated for the organic derivatization of POMs, which include (i) combination of lacunary polyoxotungstates with p-block organoderivatives;^2^ (ii) incorporation of transition metal complexes into the inorganic skeleton;^3^ and (iii) grafting of organic species through ligand condensation, as exemplified by trisalkoxo-capped Anderson–Evans polyanions.^4^ The latter represents by far the most flexible approach because it allows the incorporation of a vast number of functionalities (e.g., amines, alcohols, metal complexes) under mild synthetic conditions. Active materials for fields such as photocatalysis, photochromism or biomedicine have been prepared either by postfunctionalization of hybrid POM platforms or by direct reaction between the inorganic cluster and preformed organic moieties with the desired properties.^5,6^ Besides the classical symmetric condensation of alkoxide ligands onto both faces of Anderson–Evans polyoxomolybdates, asymmetric and single-sided derivatization have recently been achieved. Functionalization of tungstate analogues is much more complicated;^7^ in fact, reactions involving Wells–Dawson archetypes only succeed when trivanadium substituted [H_4_P_2_V_3_W_15_O_62_]^5–^ species bearing more basic O atoms are used.^8^

Among the 126 examples of trisalkoxo-functionalized isopolyoxovanadate structures registered in the CSD database,^9^ 115 correspond to Lindqvist-type POMs (Table S1). The remaining derivatives are partially (V^IV^/V^V^) or totally (V^IV^) reduced decavanadates^10^ and related species with higher nuclearity.^11^ More than 90% of those hexametalates are bicapped with trisalkoxide moieties in relative *trans* fashion and display a huge variety of pendant groups. In contrast, only two examples of *cis* derivatives have been published to our knowledge (Figure S1).^12^ Tri- and tetrafunctionalized systems have only been obtained for highly reduced vanadates,^13^ whereas isolation of monofunctionalized clusters is limited to molybdenum-substituted derivatives.^14^ Postfunctionalization has afforded systems with attractive applications in catalysis, biomedicine or electronics,^15−17^ but the vast majority of these compounds have been prepared either via complicated multistep synthesis in organic media or under harsh hydrothermal conditions. To date, only the hybrid TBA_2_[V_6_O_13_{(OCH_2_)_3_CCH_2_OH}_2_]·H_2_O (TBA = tetrabutylammonium) has been isolated from water.^18^ Encouraged by the above considerations, we decided to test the reactivity between a vanadate source and tripodal RC(CH_2_OH)_3_ molecules (R = CH_2_OH, *H*_*3*_*trisOH* and CH_3_, *H*_*3*_*trisMe*) in acidic aqueous media, in search of a direct route toward functionalized vanadate clusters. Herein, we report on the family of [H_*x*_V_7_O_18_(H_2_O)((OCH_2_)_3_CR)]^(4–*x*)–^ hybrids featuring the elusive heptavanadate cluster (Figure 1) and isolated as the following alkylammonium salts: tetramethylammonium (TMA), *x* = 2, *trisOH* (**1**) and *trisMe* (**4**); tetraethylammonium (TEA), *x* = 2.5 and *trisOH* (**2**); and ethylenediammonium (EDA), *x* = 1 and *trisOH* (**3**).

**Figure 1:**
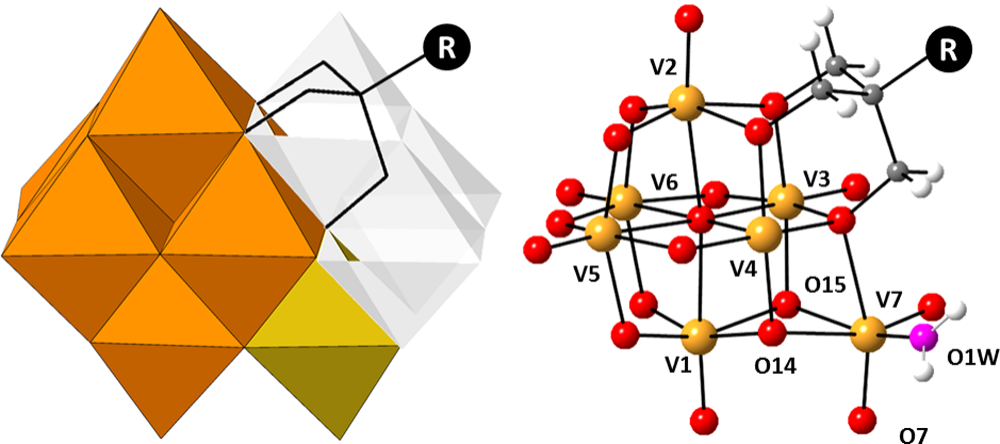
Left: polyhedral representation of [H_*x*_V_7_O_18_(H_2_O)((OCH_2_)_3_CR)]^(4–*x*)–^ hybrids (**1**–**3**, R = CH_2_OH; **4**, R = CH_3_), highlighting their structural relationship with deca- and hexavanadate anions. Color code: {VO_6_} from {V_6_O_19_} core, orange; {V(7)O_6_} center, yellow; additional {V_3_O_13_} trimer leading to {V_10_O_28_}, white. Right: ball and stick model and atom labeling for metal centers and O atoms suitable for protonation. V, orange; O, red; O1W, pink; C, gray; H, white.

First, the *trisOH* ligand was systematically reacted toward NaVO_3_ (1:2.1 molar ratio) in hot aqueous solution followed by the addition of the three distinct alkylammonium cations TMA, TEA, and EDA, and the effect of the pH was evaluated (pH = 1, 2, 3, 4, and 5). This procedure results in 15 synthetic combinations, for which all the solids originating from the slow evaporation of the final solutions were identified by FT-IR spectroscopy. Virtually identical POM regions below 1000 cm^–1^ are observed in the spectra of all the solid samples, except for those isolated from reactions carried out at pH 2 (Figures S3/S4). The former group of spectra corresponds to the organically derivatized hexavanadate anions as illustrated by a collection of six representative bands originating from different V–O vibrational modes, as well as by the signal of the C–O_POM_ stretching at 1150 cm^–1^.^19^ Fortunately, the use of TMA at pH 3 and 4 afforded single crystals, the structural elucidation of which confirmed the *trans*-bifunctionalization of Lindqvist anions in TMA_2_[V_6_O_13_{(OCH_2_)_3_CCH_2_OH}_2_] (TMA-**5**, SI). Regardless of the alkylammonium cation, reactions performed at pH 2 yielded orange crystals with similar FT-IR spectra that exhibit some differences on the characteristic signals when compared to those registered for the hybrid hexavanadates above (Figure S5).^20^

Single-crystal X-ray diffraction (Table S3) reveals that all of the three compounds TMA_2_[**1**]·4H_2_O (TMA-**1**), TEA_1.5_[**2**]·4H_2_O (TEA-**2**), and EDA_3_[**3**]_2_·2.7H_2_O (EDA-**3**) display an unprecedented monofunctionalized heptavanadate unit. The hybrid anion is best described as a central {V_6_O_19_} core with a seventh distorted {VO_5_(H_2_O)} octahedron linked to one of its triangular clefts via face-sharing. This additional center displays three terminal O atoms in *fac* configuration, one of which is a water ligand (O1W) as unequivocally established by bond valence sum calculations^21^ (BVS < 0.5). The resulting inorganic {V_7_O_21_(H_2_O)} fragment with ideal *C*_*s*_ symmetry is stabilized by the condensation of one trisalkoxo group to a single trimeric face adjacent to the seventh appended vanadate unit that is not crossed by the ideal mirror plane containing O1W, V3, V5, and V7 atoms (Figure S9). As mentioned in a previous review on polyoxovanadates,^22^ no species larger than pentamers are found to be relevant in water solution with the exception of decavanadates (Figure S10) and therefore there must be other intermediates yet to be discovered in the formation process of the latter species. In fact, the high negative charge (8−) for such a small anion could be the reason why the labile Lindqvist-type hexavanadate core has never been detected in aqueous media. The heptavanadate unit herein could well represent an intermediate species in the condensation process of the predominant decavanadate at pH = 2 that has been stabilized by the attachment of the tripodal ligand to the newly formed rhombic face, and hampers any further vanadium condensation on such cluster side (Figure 1). It is worth noting that topologically related heptaniobates [Nb_7_O_22_]^9–^ are usually found as building blocks of larger assemblies.^23^ In contrast, this work constitutes the first evidence of such elusive heptavanadate unit, in spite of the rich structural variety of low nuclearity isopolyoxovanadates covering species that range from 1 to 6 centers.^24^

The ideal *C*_s_ symmetry of the {V_7_O_21_(H_2_O)} fragment is broken down to *C*_1_ in our hybrid heptavanadates as a result of the single-sided functionalization and asymmetric protonation of O_POM_ atoms. Compounds TMA-**1** and TEA-**2** only display one of the two enantiomeric forms of the hybrid anion in their asymmetric unit, whereas EDA-**3** contains both enantiomers (**3A**/**3B**) (Figure S11). In all cases, protons are located on the trimeric face related to the functionalized region by such ideal symmetry plane, but BVS calculations show different extent of protonation for each structure. Anions **1** and **2** exhibit two protonated O atoms (μ_3_-O14/μ_2_-O15) coordinated to the same V1 center, in line with the most usual protonation sites in decavanadates. Furthermore, **2** displays an additional protonation site on the terminal O atoms of the seventh vanadium center (O7), which must show 0.5 occupation factor to obey the electroneutrality principle. In contrast, enantiomerically related **3A** and **3B** only show one proton (O14). These variations in the protonation degree of the building blocks play a key role in the crystal packing: anions showing a higher extent of protonation (**1** and **2**) interact through strong O_POM_–H···O_POM_ hydrogen bonds [2.684(7)–2.892(2) Å] established between protonated faces to arrange in dimers, whereas the hybrid units in EDA-**3** do not form any supramolecular assembly (Figure S12). This strong dimeric association in solution might prevent bifunctionalization as observed in single-sided Anderson–Evans anions.^4^ Pioneering works attributed this kind of single-sided derivatization to the aqueous media, but similar results can be obtained in protic solvents like ethanol,^5^ which allows the multiple protonation of the nonfunctionalized face.

Considering the more efficient role played by TMA as crystallizing cation, we decided to extend the studies to the *trisMe* moiety. Neither the FT-IR spectra nor the PXRD patterns of the reactions carried out at pH = 1, 3, and 4 allowed us to determine the exact nature of the resulting polycrystalline powders (Figure S7). Conversely, the pristine decavanadate anion was identified by FT-IR spectroscopy as the product of the reaction at pH = 5. In fact, the unit cell parameters of single crystals isolated from this solution agree with those reported for the Na_4_TMA_2_[V_10_O_28_]·20H_2_O phase.^25^ Reaction at pH 2 afforded crystals of TMA_2_[**4**]·5H_2_O (TMA-**4**), which shows a hybrid anion virtually identical to **1**, but for the presence of *trisMe* moiety instead. This fact confirms that (i) reactivity is highly dependent on the nature of the trisalkoxo moiety and (ii) hybrid heptavanadate anions can be isolated with different pendant functional groups.

Solution stability of molecular species is crucial for these hybrid clusters to be further involved in postfunctionalization and hence, time-resolved stability of **1** and **4** in aqueous solution was analyzed by NMR spectroscopy. No signal of the free *trisMe* group is found neither in the ^1^H NMR nor in the ^13^C NMR spectra of freshly prepared solutions of TMA-**4** (Figure S13).^26^ Nevertheless, new resonances at δ = 0.86 and 3.50 ppm, which are consistent with those of the free ligand, appear in the ^1^H NMR spectrum after 1 day. In addition, their intensity increases after 7 days, revealing the partial hydrolysis of trisalkoxo moieties (condensed/free ratio 5:1). The occurrence of such hydrolysis process is further supported by ^13^C NMR (Figure S14). In a 14 day period, 25% of the hybrids experience hydrolysis with organic groups releasing from the POM surface, after which the system reaches a dynamic equilibrium as no further hydrolysis seems to take place. Similar behavior was observed for the TMA-**1** derivative (Figures S15/S16), but the equilibrium is reached when only ca. 20% of the *trisOH* groups are released. Unlike previous observations in similar POM-based systems,^27^ addition of organic solvents to the medium does not hinder the dissociation process, as evidenced by additional experiments carried out in water/acetone (1:2) mixtures (Figure S17).

To determine whether the inorganic cluster remains stable or undergoes further transformation, we made use of a combination of ESI mass spectrometry and ^51^V NMR spectroscopy. The negative ESI mass spectrum of TMA-**4** dissolved in a H_2_O:CH_3_CN (1:1) mixture displays 10 signals in the *m*/*z* 100–600 range associated with series of intact {**4**} [V_7_O_22_C_5_H_9_ + *m*TMA^+^ + *n*H^+^ + *x*H_2_O]^(4+*m*+*n*)–^ and decavanadate {V_10_} [V_10_O_28_ + *m*TMA^+^ + *n*H^+^ + *x*H_2_O]^(6+*m*+*n*)–^ anions with different extents of protonation, counterion content, and presence of loosely associated solvent molecules.^28^ The relative intensity of the signals attributed to the {V_10_} series increases in the spectrum recorded after 1 week, and this observation, together with the NMR results mentioned above, strongly indicates that as hydrolysis of trisalkoxo ligands proceeds, the resulting heptavanadate core becomes unstable and undergoes quick reassembly into decavanadate (Figure S18). The ^51^V NMR spectrum of the freshly prepared TMA-**4** aqueous solution exhibits five resonances in the −400 to −600 ppm range that match well with the pattern of five signals with relative intensities 2:2:1:1:1 expected for **4**, in line with its ideal *C*_*s*_ symmetry if protonation (including that of O1W) is not considered. In fact, the most shielded resonance at −555 ppm might be attributed to the seventh V7 center. After 1 week, two additional signals are observed in the spectrum, which could be ascribed to the presence of the decavanadate anion as inferred from its comparison with the resonances of the Na_6_[V_10_O_28_]·18H_2_O salt registered as reference (Figure S19).

In conclusion, an unprecedented heptavanadate anion has been trapped as a series of trisalkoxide-monofunctionalized hybrids bearing two different pendant groups. Its presence in acidic solution has been rationalized as an intermediate in the quick formation of the decavanadate anion, opening the door to the detection of novel and labile species that have been predicted but never isolated to date. The extension of these studies to other polyoxometalates could also provide experimental evidence to propose potential formation mechanisms. Analysis of the solution stability of our hybrid heptavanadate anions in water shows that the hydrolysis of the organic groups reaches an equilibrium after 2 weeks and the resulting nude inorganic cores rapidly transform into decavanadate clusters. This observation indicates that our hybrid anion could well serve as POM platform for further postfunctionalization reactions.

## Supporting Information Available

Experimental section, crystallographic information, FT-IR, NMR, and ESI-MS spectra, PXRD patterns, and TGA analyses (PDF)

## Accession Codes

CCDC 2058946–2058950 contain the supplementary crystallographic data for this paper. These data can be obtained free of charge via www.ccdc.cam.ac.uk/data_request/cif, or by emailing data_request@ccdc.cam.ac.uk, or by contacting The Cambridge Crystallographic Data Centre, 12 Union Road, Cambridge CB2 1EZ, UK; fax: +44 1223 336033.
