# Supplementary material for: Isolation of the Elusive Heptavanadate Anion with Trisalkoxide Ligands
Source: Inorg Chem. 2021 Apr 5;60(8):5442–5. doi: 10.1021/acs.inorgchem.1c00448 (PMC9157487; doi:10.1021/acs.inorgchem.1c00448)
Supplement: Supplementary file 1 — ic1c00448_si_001.pdf [file ic1c00448_si_001.pdf]

Supporting Information

Isolation of the Elusive Heptavanadate Anion with Trisalkoxide Ligands

Leticia Fernández-Navarro,[‡] Aitor Nunes-Collado,[‡] Beñat Artetxe,[*,‡] Estibaliz Ruiz-Bilbao,[‡] Leire San Felices,[∥] Santiago Reinoso,[†] Ana San José Wéry,[⊥] and Juan M. Gutiérrez–Zorrilla[*,‡,§]

[‡]Departamento de Química Inorgánica, and [∥]Servicios Generales de Investigación SGIker, Facultad de Ciencia y Tecnología, Universidad del País Vasco UPV/EHU, P.O. Box 644, 48080 Bilbao, Spain.

[†]Departamento de Ciencias and Institute for Advanced Materials and Mathematics (InaMat[2]), Universidad Pública de Navarra (UPNA), Campus de Arrosadia, 31006 Pamplona, Spain.

[⊥]Departamento de Desarrollo Sostenible, Universidad Católica de Ávila, c/Canteros s/n, 05005 Ávila, Spain.

[§]BCMaterials, Basque Center for Materials, Applications and Nanostructures, UPV/EHU Science Park, 48940 Leioa, Spain.

Table S1. List of polyoxovanadates bearing tripodal organic groups deposited in the CSD database. 3

Figure S1. Commonly observed functionalization modes for Anderson-Evans polyoxomolybdates (δ isomer) and Lindqvist-type hexavanadate anions with trisalkoxide ligands..4

1.- EXPERIMENTAL SECTION 5

Materials and Methods. 5

General Synthetic Procedure. 5

Thermal Analyses. 6

Figure S2. TGA/DTA curves for compounds **TMA-1**, **TEA-2**, **EDA-3**, **TMA-4** and **TMA-5**. 6

Table S2. Experimental values of the thermogravimetric analyses of **TMA-1**, **TEA-2**, **EDA-3**, **TMA-4** and **TMA-5**, compared to calculated values for each mass loss step..7

Single-Crystal X-Ray Diffraction. 7

Table S3. Crystallographic Data for **TMA-1, TEA-2, EDA-3, TMA-4** and **TMA-5.** 8

FT-IR Spectroscopy and PXRD Analyses.. 8

Figure S3. FT-IR spectra in the 1000-400 cm^{-1} region of the solid products obtained from the reaction of $NaVO_3$ with different tripodal ligands and alkylammonium salts in acidic aqueous solution. 9

Figure S4. Expanded FT-IR spectra in the 2000 to 400 cm^{-1} region for compounds **TMA-1**, **TEA-2**, **EDA-3** and **TMA-4** (dark blue spectra in Figure S2) together with the assignment of the key signals to confirm the successful organic functionalization. 11

Figure S5. Comparison of the inorganic regions in the FT-IR spectra of $Na_4TMA_2[V_{10}O_{28}]\cdot 20H_2O$ (pink), **TMA-5** (green) and **TMA-1** (dark blue)..11

Figure S6. PXRD patterns of compounds **TMA-1**, **TEA-2**, **EDA-3** and **TMA-4** compared with those simulated from their respective single-crystal X-ray diffraction data. 12

Figure S7. PXRD patterns of the different solid samples obtained from the reaction among $NaVO_3$, the *H_3trisMe* and TMACl at different acidic pH values 13

Figure S8. Summary of the results obtained from the reactions between $NaVO_3$, two different tripodal ligands and three different alkylammonium salts as a function of the pH (code: X, non-identified solid residue; $(trisOH)_2V_6$, samples that display the same hybrid POM as **TMA-5** according to their FT-IR spectra, but could not be analyzed by single-crystal X-ray diffraction).. 13

2.- CRYSTAL STRUCTURE 14

Figure S9. Illustration of the ideal symmetry plane of the inorganic $\{V_7O_{22}(H_2O)\}$ cluster depicted over the crystal structure of **TMA-4** in polyhedral (left) and ball and stick (right) representation. 14

Figure S10. Structure of predominant polyoxovanadate species in aqueous solution as a function of pH. Note that octahedral vanadium centers are observed at acidic pH, whereas species formed at basic pH are constituted by tetrahedral units 14

Figure S11. Polyhedral and ORTEP view showing 50% probability ellipsoids of the hybrid heptavanadate units in **TMA-1**, **TEA-2**, **EDA-3**, **TMA-4** and **TMA-5**, together with atom labelling scheme. 15

Table S4. V—O bond lengths (Å) in compounds **TMA-1**, **TEA-2**, **EDA-3**, **TMA-4** and **TMA-5** 16

Table S5. BVS values in **TMA-1**, **TEA-2**, **EDA-3** and **TMA-4** (protonated positions highlighted in bold). 16

Crystal Packing 16

Figure S12. View of the crystal packing of a) **TMA-1**, b) **TEA-2**, c) **EDA-3**, d) **TMA-4** and e) **TMA-5**. 17

Table S6. Donor···Acceptor Distances (Å) for the Hydrogen Bond Interactions in **TMA-1**. 18

Table S7. Donor···Acceptor Distances (Å) for the Hydrogen Bond Interactions in **TEA-2**. 18

Table S8. Donor···Acceptor Distances (Å) for the Hydrogen Bond Interactions in **EDA-3**. 19

Table S9. Donor···Acceptor Distances (Å) for the Hydrogen Bond Interactions in **TMA-4.** 20

Table S10. Donor···Acceptor Distances (Å) for the Hydrogen Bond Interactions in **TMA-5.** 20

3.- SOLUTION STABILITY 21

Table S11. Integration of the ^{1}H-RMN signals for an aqueous solution of **TMA-4** after 0, 1, 7, 14 and 21 days referenced to that of TMA cations (24 H, s, 3.25 ppm) and compared to free H_3*trisMe*. 21

Figure S13. ^{1}H-RMN spectrum of a freshly prepared aqueous solution of **TMA-4** together with those recorded after 1, 7, 14 and 21 days and compared to that of the free H_3*trisMe* ligand provided as reference. 22

Table S12. ^{13}C-RMN signals of an aqueous solution of **TMA-4** after 0 and 7 days compared to those of H_3*trisMe*...23

Figure S14. ^{13}C-RMN s spectrum of a freshly prepared aqueous solution of **TMA-4** together with that recorded after 7 days and compared to that of the free H_3*trisMe* ligand provided as reference. 23

Table S13. Integration of the ^{1}H-RMN signals for an aqueous solution of **TMA-1** after 0, 1, 7, 14 and 21 days referenced to that of TMA cations (24H, s, 3.24 ppm) and compared to free H_3*trisOH*. 24

Figure S15. ^{1}H-RMN spectrum of a freshly prepared aqueous solution of **TMA-1** together with those recorded after 1, 7, 14 and 21 days and compared to that of the free H_3*trisOH* ligand provided as reference. 25

Table S14. ^{13}C-RMN signals of an aqueous solution of **TMA-1** after 0 and 7 days compared to those of H_3*trisOH*.26

Figure S16. ^{13}C-RMN spectrum of a freshly prepared aqueous solution of **TMA-1** together with that recorded after 7 days and compared to that of the free H_3*trisOH* ligand provided as reference. 26

Figure S17. ^{1}H-RMN spectrum recorded after 14 days of a sample of **TMA-4** dissolved in a mixture of acetone:water (2:1) 27

Figure S18. ESI-MS spectra of a freshly prepared sample of **TMA-4** in water:acetonitrile (1:1) and that recorded after one week. Abbreviations: $\{$**4+TMA**$\}^{4-}$= $[V_7O_{22}C_5H_9$ + TMA + H + $6H_2O]^{4-}$; $\{$**4**$\}^{4-}$= $[V_7O_{22}C_5H_9$ + 2H + $H_2O]^{4-}$; $\{$**4+TMA**$\}^{3-}$= $[V_7O_{22}C_5H_9$ + TMA + 2H + $4H_2O]^{3-}$; $\{$**4**$\}^{2-}$= $[V_7O_{22}C_5H_9$ + 4H + $8H_2O]^{2-}$; $\{$**V$_{10}$**$\}^{5-}$= $[V_{10}O_{28}$ + H + $3H_2O]^{5-}$; $\{$**V$_{10}$+TMA**$\}^{4-}$= $[V_{10}O_{28}$ + TMA + H + $5H_2O]^{4-}$; $\{$**V$_{10}$+TMA**$\}^{3-}$= $[V_{10}O_{28}$ + TMA + 2H + $3H_2O]^{3-}$; $\{$**V$_{10}$+2TMA**$\}^{3-}$= $[V_{10}O_{28}$ + 2TMA + H + $2H_2O]^{3-}$; $\{$**V$_{10}$+TMA**$\}^{2-}$= $[V_{10}O_{28}$ + TMA + 3H + $4H_2O]^{3-}$; $\{$**4+2TMA**$\}^{4-}$= $[V_7O_{22}C_5H_9$ + 2TMA$]^{4-}$; $\{$**V$_{10}$+TMA**$\}^{5-}$= $[V_{10}O_{28}$ + TMA + $5H_2O]$ 28

Figure S19. ^{51}V-NMR spectrum of a freshly prepared aqueous solution of **TMA-4** together with that recorded after 7 days and compared to that of the $Na_6[V_{10}O_{28}]\cdot 18H_2O$ provided as reference. 29

Table S1. List of polyoxovanadates bearing tripodal organic groups deposited in the CSD database.

Refcode	Formula	Nº Funct.	Oxid. st.
QOTZAD	$(TBA)_2[V^{V}_6O_{13}\{(OCH_2)_3CCH_2OOCCH_2CH_2CONHCH_2COOCH_2CH_3\}_2]$	2 Trans	Ox.
ACIJEE	$(TBA)_2[V^{V}_6O_{13}\{(OCH_2)_3CCH_2OCH_2C\equiv CH\}_2]$	2 Trans	Ox.
ACIJII	$(TBA)_2[V^{V}6O_{13}\{(OCH_2)_3CCH_2OCH_2CCHN_3CH_2CO_2CH_2CH_3\}_2]$	2 Trans	Ox.
BIYPUX	$(TBA)_2[V^{V}_6O_{13}\{(OCH_2)_3CCH_2OCOCH_2CH_2CONHCH_2CH_2COOCH_3\}_2]$	2 Trans	Ox.
COSRIN	$(TBA)_6[V^{V}_6O_{13}\{(OCH_2)_3CNMo_6O_{18}\}_2]\cdot CH_3CN$	2 Trans	Ox.
DEBZES	$Na_2[V^{IV}_6O_7\{(OCH_2)_3CNH_2\}_4]$	4	Red.
EGEMUZ	$[TBA)_2[V^{V}_6O_{13}\{(OCH_2)_3CCH_2OCO(4\text{-}C_5H_4N)\}_2]$	2 Trans	Ox.
EGENEK	$(TBA)_2[V^{V}_6O_{13}\{(OCH_2)_3CNHCO(4\text{-}C_5H_4N)\}_2]$	2 Trans	Ox.
EMIFEM	$(TBA)_2\{V^{V}_6O_{13}(OCH_2)_3CCH_2OOC(CH_2)_{16}CH_3\}_2]$	2 Trans	Ox.
EMIFEM01	$(TBA)_2\{V^{V}_6O_{13}(OCH_2)_3CCH_2OOC(CH_2)_{16}CH_3\}_2]$	2 Trans	Ox.
FOMKIE	$(TBA)_2[V^{V}_6O_{13}\{(OCH_2)_3C\text{-}CH_2OOCCH_2CH_2CONHCH(COOC(CH_3)_3)CH_2CH(CH_3)_2\}_2]$	2 Trans	Ox.
GIFROE	$(TBA)_2[V^{V}_6O_{13}\{(OCH_2)_3CCH_2OH\}_2\cdot H_2O$	2 Trans	Ox.
GIFRUK	$(TBA)_2[V^{V}_6O_{13}\{(OCH_2)_3CCH_2OH\}_2\cdot 4CH_3CN$	2 Trans	Ox.
GIXTEP	$(TBA)_2[V^{V}_6O_{13}\{(OCH_2)_3CCH_2OOCCH_2CH_2CONHCH_2CH_2COOCH_2CH_3\}_2]$	2 Trans	Ox.
GIXTIT	$(TBA)_2[V^{V}_6O_{13}\{(OCH_2)_3CCH_2OOCCH_2CH_2CONHCH(CH_3)COOCH_3\}_2]$	2 Trans	Ox.
GOPQUZ	$(TBA)_2[V^{V}_6O_{13}(OCH_2)_3CNH_2(OCH_2)_3CNHCH_2C_6H_4COOC_{16}H_{33}]$	2 Trans	Ox.
HAHME	$Ba[V^{IV}_6O_7(OH)_3\{((OCH_2)_3CCH_3]_3)\cdot 3H_2O$	3	Red.
HAHMIM	$Rb_2[V^{IV}_6O_7(OH)_3((OCH_2)_3CCH_3)_3$	3	Red.
INEMEV	$(TBA)_2[V^{V}_6O_{13}\{(OCH_2)_3CNHCH_2C_{16}H_9\}_2]$	2 Trans	Ox.
JODLAS	$(TBA)_2[V^{V}_6O_{13}\{(OCH_2)_3CCH_2OOCCH_2CH_2CONHCH(COOCH_3)CH_2CH(CH_3)_2\}_2]\cdot[(CH_3CH_2)_2O]$	2 Trans	Ox.
JODLOG	$(TBA)_2[V^{V}_6O_{13}\{(OCH_2)_3CCH_2OOCCH_2CH_2CONHCH(COOCH_3)CH_2CH(CH_3)_2\}_2]\cdot[(CH_3CH_2OH]$	2 Trans	Ox.
JOXCII	$((CH_3)_3NH)[V^{IV}_5V^{V}O_7(OH)_3\{(CH_2O_3)CCH_3\}_3]$	3	Part. red.
JOXCOO	$Na_2[V^{IV}_6O_7\{(CH_2O)_3CCH_2CH_3\}_4]$	4	Red.
JOXCII10	$((CH_3)_3NH)[V^{IV}_5V^{V}O_7(OH)_3\{((OCH_2)_3CCH_3\}_3]$	3	Part. red.
JOXCOO10	$Na_2[V^{IV}_6O_7\{(CH_2O)_3CCH_2CH_3\}_4]$	4	Red.
KOSMOU	$(NH_4)_4[V^{IV}_{10}O_{16}\{(CH_2O)_3CCH_2CH_3\}_4]\cdot 4H_2O$	4	Red.
KOSMOU10	$(NH_4)_4[V^{IV}_2O_{16}\{(CH_2O)_3CCH_2CH_3\}_4]\cdot 4H_2O$	4	Red.
KURBEE	$(TBA)_2[V^{V}_6O_{13}\{(OCH_2)_3CNHCOCH=CH_2\}_2]$	2 Trans	Ox.
KURBII	$(C_5H_5NH)_2[V^{V}_6O_{13}\{(OCH_2)_3CCH_3\}_2]\cdot 2CH_3NCOH$	2 Trans	Ox.
KURBOO	$(TBA)_2[V_3{}^{V}V_3{}^{IV}O_{10}(OH)_3\{(OCH_2)_3CNO_2\}_2]\cdot 0.67H_2Cl_2$	2 Trans	Part. red.
KURBUU	$(TBA)_2[V_6{}^{IV}O_{10}(OH)_6\{(OCH_2)_3CCH_3\}_2]\cdot 2HNPhNHPh$	2 Trans	Red.
LEVGOI	$[V^{IV}_{16}O_{20}\{(OCH_2)_3CH_2H\}_8(H_2O)_4]\cdot 3H_2O$	8	Red.
LIZYEB	$(TBA)_2[V^{V}_6O_{13}\{(OCH_2)_3CCH_2OOCCH_2CH_2COON(C=O)_2(CH_2)_2\}_2]$	2 Trans	Ox.
LOFJAU	$(TBA)_2[V^{V}_6O_{13}\{(OCH_2)_3CCH_2OOCCH_2CH_2CONHCH_2COOC(CH_3)_3\}_2]$	2 Trans	Ox.
LOFJEY	$(TBA)_2[V^{V}_6O_{13}\{(OCH_2)_3CCH_2OOCCH_2CH_2CONHCH(CH_3)COOC(CH_3)_3\}_2]$	2 Trans	Ox.
LOFVUY	$Tb(C_{10}H_8N_2O_2)_2[V^{V}_6O_{13}\{(OCH_2)_3C(NH_2C_7H_6\text{-}4\text{-}CO_2)\}\{(OCH_2)_3C(NHC_7H_6\text{-}4\text{-}CO_2)\}_2]\cdot 0.4DMF$	2 Trans	Ox.
LOFVUY01	$Tb(C_{10}H_8N_2O_2)_2[V^{V}_6O_{13}\{(OCH_2)_3C(NH_2C_7H_6\text{-}4\text{-}CO_2)\}\{(OCH_2)_3C(NH\ C_7H_6\text{-}4\text{-}CO_2)\}_2]\ 0.4DMF$	2 Trans	Ox.
LOFWAF	$(TBA)_2[V^{V}_6O_{13}\{(OCH_2)_3C(NHCH_2C_6H_4\text{-}4\text{-}CO_2H)\}_2]$	2 Trans	Ox.
MAGZAY	$(TBA)_2[V^{V}_6O_{13}\{(OCH_3)_3CCH_2OOCCH_2CH_2CONHCH_2CH_2CH_2CH_3\}_2]$	2 Trans	Ox.
MAGZEC	$(TBA)_2[V^{V}_6O_{13}\{(OCH_3)_3CCH_2OOCCH_2CH_2\ CONHCH_2CH=CH_2\}_2\ ]$	2 Trans	Ox.
MAGZIG	$(TBA)_2[V^{V}_6O_{13}\{(OCH_3)_3CCH_2OOCCH_2CH_2CONHCH_2CH_2CH_3\}_2]$	2 Trans	Ox.
MAGZOM	$(TBA)_2[V^{V}_6O_{13}\{(OCH_3)_3CCH_2OOCCH_2CH_2COOH\}_2\ ]$	2 Trans	Ox.
NAPWAF	$(TBA)_2[V^{V}_6O_{13}\{(OCH_2)_3CCOO(CH_2)12OH\}_2]\cdot 2CH_3CH_2OH$	2 Trans	Ox.
NAPWEJ	$(TBA)_2[V^{V}_6O_{13}\{(OCH_2)_3CCOO(CH_2CH_2O)_3CH_3\}_2]$	2 Trans	Ox.
NAPWIN	$(TBA)_2[V^{V}_6O_{13}\{(OCH_2)_3CCOO(CH_2)_8CH=CH_2\}2$	2 Trans	Ox.
NAPWOT	$(TBA)_2[V^{V}_6O_{13}\{(OCH_2)_3CCOO(CH_2)_{15}CH_3\}_2]$	2 Trans	Ox.
NAPWUZ	$(TBA)_2[V^{V}_6O_{13}\{(OCH_2)_3CCOOCH_2CF_3\}_2]$	2 Trans	Ox.
NEGROJ	$(TBA)_2[V^{V}_6O_{13}\{(OCH_2)_3CCH_2OOCCH_2CH_2CON(C_6H_{11})CONHC_6H_{11}\}_2]$.	2 Trans	Ox.
NEMDEP	$(TBA)_2[V^{V}_6O_{13}\{(OCH_2)_3C(4\text{-}CONHC_5H_4N)\}_2]\ 1.8DMF$	2 Trans	Ox.
NEMDIT	$[Co(H_2O)_2(DMF)_2(V^{V}_6O_{13}\{(CH_2O)_3CNHCO(4\text{-}C_5H_4N)\}_2)]_n$	2 Trans	Ox.
NEMDOZ	$[Mn(H_2O)_2(DMF)_2(V^{V}_6O_{13}\{(CH_2O)_3CNHCO(4\text{-}C_5H_4N)\}_2)]_n$	2 Trans	Ox.
NEMDUF	$[Ni(H_2O)_2(DMF)_2(V^{V}_6O_{13}\{(CH_2O)_3CNHCO(4\text{-}C_5H_4N)\}_2)]_n$	2 Trans	Ox.
NEMFAN	$[Zn(H_2O)_2(DMF)_2(V_6O_{13}\{(CH_2O)_3CNHCO(4\text{-}C_5H_4N)\}_2)]_n$	2 Trans	Ox.
OFOTEL	$(TBA)_2[V^{V}_6O_{13}\{(OCH_2)_3CCH_2OCH_2\text{-}4\text{-}C_6H_4I\}_2]\cdot CH_3CN$	2 Trans	Ox.
OFOTIP	$(TBA)_2[V^{V}_6O_{13}\{(OCH_2)_3CCH_2OCH_2\text{-}4\text{-}C_6H_4CH=CHCON(CH_3)_2\}_2]\cdot CH_2Cl_2$	2 Trans	Ox.
OQETOU	$(TBA)_2[V^{V}_6O_{13}\{(OCH_2)_3CCH_2OOCCH_2CH_3\}_2]$	2 Trans	Ox.
OQETUA	$(TBA)_2[V^{V}_6O_{13}\{(OCH_2)_3CCH_2OOC(CH_2)_4CH_3\}_2]$	2 Trans	Ox.
OQETOU01	$(TBA)_2[V^{V}_6O_{13}\{(OCH_2)_3CCH_2OOCCH_2CH_3\}_2]$	2 Trans	Ox.
PAGMUF	$(TEA)[V^{IV}_{10}O_{13}\{(CH_3CH_2C(CH_2O)_3\}_5$	5	Red.
PAGNAM	$(TMA)_{2/3}(TEA)_{1/3}[V^{IV}_{10}O_{13}\{CH_3C(CH_2O)_3\}_5]\cdot 0.69H_2O$	5	Red.
PAGSOF	$[V^{V}_6O_{11}(OH)_2\{(OCH_2)_3CCH_3\}_2]\cdot CH_3CH_2O\cdot 3HOCN(CH_3)_2$	2 Trans	Ox.
PAGSUL	$(TBA)_2[V^{IV}_4V^{V}_2O_9(OH)_4\{(OCH_2)_3CCH_3\}_2]$	2 Trans	Part. red.

PAGTAS	$(TBA)_2[V^{IV}_6O_7(OH)_6\{(OCH_2)_3CCH_3\}_2]\cdot 2CH_2Cl_2\cdot 0.5C_{12}H_{10}N_2$	2 Trans	Red.
PAVSOX	$(NH_4)_3[V^{IV}_6O_8(OH)_2\{(OCH_2)_3CCH_3\}_3]$	3	Red.
PAVSUD	$Na_2[V^{IV}_6\ O_7(OH)_3\{(OCH_2)_3CCH_2OH\}_3]\cdot 9H_2O$	3	Red.
PAVTAK	$Na_2[V^{IV}_3V^{V}_3O_{10}\{(OCH_2O)CNH_2\}_3]\cdot 9H_2O$	3	Part. red.
PAVTEO	$(NH_4)_2[V^{IV}_6O_7(OH)_3\{(OCH_2)_3CC_2H_5\}_3]\cdot 4H_2O$	3	Red.
PAVTIS	$Na_{0.5}(TBA)_{1.5}[V^{IV}_3V^{V}_3O_{10}\{(OCH_2)_3CNHCOC_6H_5\}_3]\cdot H_2O\cdot 3C_2H_5OH$	3	Part. red.
PIQGEB	$(NH_4)_7[H_7V^{IV}_{12}V^{V}_7O_{50}(CH_2)_3CCH_2OH]\cdot 11.5\ H_2O$	1	Part. red.
QIPHEF	$(CH_3CH_2NH_2)[V^{V}_6O_{13}\{(OCH_2)_3CCH_2OOCCH_2CH_2CONHC_6H_4Br\text{-}\mathit{p})\}_2]$	2 Trans	Ox.
QIPHIJ	$(CH_3CH_2NH_2)_2[V^{V}_6O_{13}\{(OCH_2)_3CCH_2OOCCH_2CH_2CONHC_6H_4I\text{-}\mathit{p})\}_2]$	2 Trans	Ox.
QIPHOP	$(CH_3CH_2NH_2)_2[V^{V}_6O_{13}\{(OCH_2)_3CCH_2OOCCH_2CH_2CONHC_6H_4Cl\text{-}\mathit{p})\}_2]\cdot 2CH_3CN$	2 Trans	Ox.
REFCIR	$(TBA)_2[V^{V}_6O_{13}\{(OCH_2)_3CCH_2SO_3C_7H_4\}_2]\cdot 2.5CH_3CN$	2 Trans	Ox.
REFCOX	$(TBA)_2[V^{V}_6O_{13}\{(OCH_2)_3CCH_2N_3\}_2]\cdot 4CH_3CN$	2 Trans	Ox.
REFLEW	$(TBA)_2[V^{V}_6O_{13}\{(OCH_2)_3CCH_2Br\}_2]$	2 Trans	Ox.
RIXJOA	$(TBA)_2[V^{V}_6O_{13}\{(OCH_2)_3CCHCH_3SCH_3\}2]\cdot 2CH_2Cl_2$	2 Trans	Ox.
RIXJUG	$(TBA)_2[V^{V}_6O_{13}\{(OCH_2)_3CCH_2SCH_3\}2]\cdot 1.5CH_2Cl_2$	2 Trans	Ox.
RIXKAN	$(TBA)_2[V^{V}_6O_{13}\{(OCH_2)_3CCH_2SPh\}_2]$	2 Trans	Ox.
RIXKER	$(TBA)_2[V^{V}_6O_{13}\{(OCH_2)_3CCH_2S_2\text{-}C_{10}H_7\}_2]$	2 Trans	Ox.
RIXKIV	$(TBA)_2[V^{V}_6O_{13}\{(OCH_2)_3CCH_2SCF_3\}_2]\cdot 0.75CH_2Cl_2$	2 Trans	Ox.
RIXKOB	$(TBA)_2[V^{V}_6O_{13}\{(OCH_2)_3CCH_2SCH_3\}_2]\cdot 0.80CH_2Cl_2\cdot 0.8(CH_3CH_2)_2O$	2 Trans	Ox.
SAJWEH	$[V^{V}_6O_{13}\{(OCH_2)_3CNH_3\}]\ 2.4DMSO$	2 Trans	Ox.
SAJWIL	$(TBA)_2[V^{V}_6O_{13}\{(OCH_2)_3C(NH(CO)CH_2CH_2CH_2C_{16}H_9)\}\{(OCH_2)_3CNH_2\}]$	2 Trans	Ox.
SAJWOR	$(TBA)_2[V^{V}_6O_{13}\{(OCH_2)_3CNHCOCH_2CH_2CH_2C_{16}H_9)\}_2]$	2 Trans	Ox.
SAMMOK	$(TBA)_2[V^{V}_6O_{13}\{(OCH_2)_3CNHCO\text{-}3\text{-}C_5H_4N\}_2]$	2 Trans	Ox.
SEVPER	$(TBA)_2[V^{V}_6O_{13}\{(OCH_2)_3CCH_2OOC(C_6H_4)CONHCH_2CH_2CH_3\}_2]$	2 Trans	Ox.
SEVPIV	$(TBA)_2[V^{V}_6O_{13}\{(OCH_2)_3CCH_2OOC(C_6H_4)CONHCH_2CH{=}CH_2\}_2]$	2 Trans	Ox.
SEVPOB	$(TBA)_2[V^{V}_6O_{13}\{(OCH_2)_3CCH_2OOC(C_6H_4)CONHCH_2CH_2CH_2CH_3\}_2]$	2 Trans	Ox.
SEVQUI	$(TBA)_2[V^{V}_6O_{13}\{(OCH_2)_3CCH_2OOCC_6H_4CONHPh\}_2]$	2 Trans	Ox.
SIDQII	$(TBA)Ag[V^{V}_6O_{13}\{(OCH_2)_3NC_5H_4\text{-}4\text{-}CH_3\}_2]$	2 Trans	Ox.
SIDZOX	$TBA_2[V^{V}_6O_{13}\{(OCH_2)_3\text{-}NC_5H_4\text{-}4\text{-}CH_3\}_2]\cdot 2CH_3CN$	2 Trans	Ox.
SIDZUD	$(TBA)_3Cu[V^{V}_6O_{13}\{(OCH_2)_3\text{-}NC_5H_4\text{-}4\text{-}CH_3\}_2]_2\cdot 4DMF$	2 Trans	Ox.
TEKKIG	$(TBA)_2[V^{V}_6O_{13}\{(OCH_2)_3C_7H_8N_3O_2\}_2]\cdot 2CH_3CN$	2 Trans	Ox.
TEKKOM	$(TBA)_2[V^{V}_6O_{13}\{(OCH_2)_3C_5H_6N_3O\}_2]\cdot 1.5CH_3CN$	2 Trans	Ox.
TEKLON	$(TBA)_2[V^{V}_6O_{13}\{(OCH_2)_3C_{10}H_7N_3Cl\}_2]$	2 Trans	Ox.
TEKLUT	$(TBA)_2[V^{V}_6O_{13}\{(OCH_2)_3C_{11}H_{10}N_3\}_2]$	2 Trans	Ox.
UCOTEN	$(TBA)_2[V^{V}_6O_{13}\{(OCH_2)_3NHCOC_{14}N_3H_{10}\}_2]$	2 Trans	Ox.
VERDAX	$(TBA)_2[V^{V}_6O_{13}\{(OCH_2)_3CNO_2\}_2]$	2 Trans	Ox.
VERDAX10	$(TBA)_2[V^{V}_6O_{13}\{(OCH_2)_3CNO_2\}_2]$	2 Trans	Ox.
VERDEB	$(TBA)_2[V^{V}_6O_{13}\{(OCH_2)_3CCH_3\}_2]$	2 Trans	Ox.
VERDEB10	$(TBA)_2[V^{V}_6O_{13}\{(OCH_2)_3CCH_3\}_2]$	2 Trans	Ox.
VERDIF	$(TBA)_2[V^{V}_6O_{13}\{(OCH_2)_3CCH_2OH\}_2]$	2 Trans	Ox.
VERDIF10	$(TBA)_2[V^{V}_6O_{13}\{(OCH_2)_3CCH_2OH\}_2]$	2 Trans	Ox.
VERKOV	$(C_8H_{20}N_3O)_2[V^{V}_6O_{13}\{(OCH_2)_3CCH_2OH\}_2]\cdot CH_2Cl_2$	2 Trans	Ox.
WAFVUW	$(TBA)_2[\{FcCONHC(CH_2O)_3\}_2V^{V}_6O_{13}]\cdot 2C_3H_7NO$	2 Trans	Ox.
WAGSUV	$(TBA)_2[V^{V}_6O_{13}\{(OCH_2)_3CNC_8H_8O\}_2]$	2 Trans	Ox.
WAGTAC	$[V^{V}_6O_{13}\{(OCH_2)_3CNH_3\}_2]\cdot 4H_2O$	2 Trans	Ox.
XIHGAX	$[Co_2(C_{10}H_4N_2)_2(DMSO)_2(DMF)_2(H_2O)_2V^{V}_6O_{13}\{(CH_2O)_3CNHCH_2(4\text{-}C_6H_4COO)\}_2]\cdot 2.1DMF$	2 Trans	Ox.
YADLAT	$(TBA)_2[V^{V}_6O_{13}\{(OCH_2)_3CCH_2OOCC(CH_3){=}CH_2\}_2]$	2 Trans	Ox.
YAFROP	$(TBA)_2[V^{V}_6O_{13}\{(OCH_2)_3CCH_2OOC(C_6H_4)COOH\}_2$	2 Trans	Ox.
YAFRUV	$(TBA)_2[V^{V}_6O_{13}\{(OCH_2)_3CCH_2OOC(C_6H_8)COOH\}_2$	2 Trans	Ox.
ZAZYIL	$(TBA)_2[V^{V}_6O_{13}\{(OCH_2)_3CCH_2OOCC_6H_4Br\text{-}\mathit{p}\}_2]$	2 Trans	Ox.
ZAZYIL	$(TBA)_2[V^{V}_6O_{13}\{(OCH_2)_3CCH_2OOCC_6H_4NO_2\text{-}\mathit{m}\}_2]$	2 Trans	Ox.
ZAZYUX	$(TBA)_2[V^{V}_6O_{13}\{(OCH_2)_3CCH_2OOCC_4H_3S\}_2]$	2 Trans	Ox.
ZEVLUH	*cis*-$Na_2[V^{IV}_6O_7(OH)_6\{(OCH_2)_3CCH_2OH\}_2]\cdot 8H_2O$	2 cis	Red.
ZEVMAO	*cis-*$(CN_3H_6)_3[V^{IV}V^{V}_5O_{13}\{(OCH_2)_3CCH_2OH\}_2]\cdot 4.5H_2O$	2 cis	Part. red.
ZEVMES	*trans*-$(CN_3H_6)_2[V^{V}_6O_{13}\{(OCH_2)_3CCH_2OH\}_2]\cdot H_2O$	2 Trans	Ox.
KUGHOM	$[C_{24}H_{20}P]_2[V^{V}_6O_{13}\{C_{61}H_{58}N_5O_4ZnONC_3H_7\}_2]$	2 Trans	Ox.
KUKYOH	$[(C_{18}H_{16}BF_2I_2N_3)(C_6H_{13})]_2[V^{V}_6O_{13}\{(OCH_2)_3CCH_2OH\}_2]\cdot 2H_2O$	2 Trans	Ox.
WUKVUW	$(C_8H_{19}N)_2[V^{V}_6O_{13}\{(OCH_2)_3CNHCOC_6H_4(OC_{12}H_{25})\text{-}p\}_2$	2 Trans	Ox.
WULHIX	$(C_8H_{19}N)_2[V^{V}_6O_{13}\{(OCH_2)_3CNHCOC_6H_3(OC_{12}H_{25})_2\text{-}m_2\}_2]\cdot 2Dmac$	2 Trans	Ox.
WULHOD	$(C_8H_{19}N)_2[V^{V}_6O_{13}\{(OCH_2)_3CNHCOC_6H_4(OC_{16}H_{33})\text{-}p\}_2]\cdot 2CH_3CN$	2 Trans	Ox.

Abbreviations: Refcode = CSD refcode; Nº Funct. = number of organic functionalities; Oxid. st. = oxidation states of V centers (Ox. = fully oxidized V^{V}; Red. = totally reduced V^{IV}; Part. red. = partially reduced, mixed-valence V^{IV}/V^{V}); Dmac: dimethylacetamide.

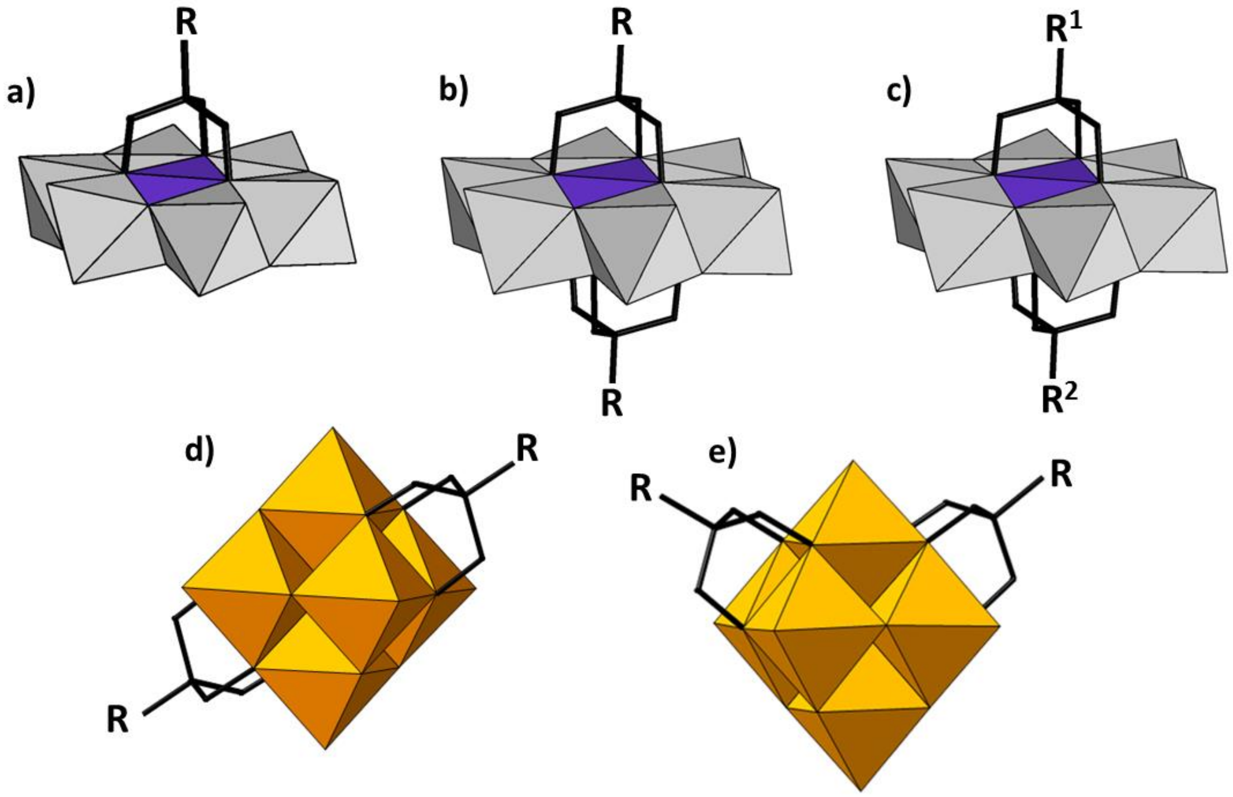

Figure S1. Commonly observed functionalization modes for Anderson-Evans polyoxomolybdates (δ-isomer) and Lindqvist-type hexavanadate anions with trisalkoxide ligands. a) Single-sided, b) symmetric, double-sided and c) asymmetric, double-sided functionalization of Anderson-Evans anions; d) *trans*- and e) *cis*-isomers of bicapped hexavanadate units.

1.- EXPERIMENTAL SECTION

Materials and Methods. All chemicals were obtained from commercial sources and used without further purification. Carbon, hydrogen and nitrogen contents were determined on a Perkin–Elmer 2400 CHN analyzer and infrared spectra (FT-IR) were recorded as KBr pellets on a SHIMADZU FTIR–8400S spectrophotometer. Each spectrum was recorded in the 400-4000 cm^{-1} region with a resolution of 4 cm^{-1}. Powder X-ray diffraction (PXRD) patterns were recorded in the $5 \leq 2\theta \leq 35°$ range (0.033° step size, 30 s per step) using a Philips X'PERT PRO diffractometer (40 kV/40 mA, θ–θ configuration) equipped with monochromated CuKα radiation (λ = 1.5418 Å) and a PIXcel detector. The ^{1}H-, ^{13}C- and proton-decoupled ^{51}V-NMR spectra were acquired for a 10^{-2} M solution of the corresponding compound (**TMA-1** or **TMA-4**) in deuterated water (D_2O) or water (pH = ca. 4) on a Bruker AC-500 spectrometer (500 MHz for ^{1}H, 125.7 MHz for ^{13}C and 52.6 MHz for ^{51}V). Spectra were referenced to external tetramethylsilane, sodium metavanadate and the residual protonated solvent for ^{1}H-NMR. All chemical shifts (δ) are reported in parts per million (ppm). Electrospray ionization mass spectra (ESI-MS) were obtained on a sample of **TMA-4** dissolved in a 1:1 water:acetonitrile mixture with concentration of 10^{-5} M. The solution was introduced at a flow rate of 10 $\mu L \cdot min^{-1}$ in a Waters QTOF Premier instrument operating in the negative scan mode (V-mode) with orthogonal Z-spray electrospray interface, capillary voltage of 3.3 kV, N_2 as desolvation (300 $L \cdot h^{-1}$) and cone gas (30 $L \cdot h^{-1}$), typical desolvation (200 °C) and source block (120 °C) temperatures, and low cone voltage (U_c) of 15 V to control the extent of fragmentation of the gas-phase detected species.

General Synthetic Procedure. $NaVO_3$ (0.512 g, 4.2 mmol) was dissolved in 25 mL of water and the pH of the solution was adjusted to 2 using aqueous 4M HCl. Then, the trisalkoxide molecule [2 mmol; $CH_3C(CH_2OH)_3$ (*H_3trisMe*): 0.240 g; $C(CH_2OH)_4$ (*H_3trisOH*): 0.272 g] was added and the solution was heated to 90 °C for 1 h. Finally, the corresponding alkylammonium salt [5 mmol; $[(CH_3)_4N]Cl$ (TMACl): 0.550 g; $[(CH_3CH_2)_4N]Cl$ (TEACl): 0.826 g; $[H_3N(CH_2)_2NH_3]Cl_2$ ($EDACl_2$): 0.665 g] was added and the resulting orange solution was left to cool down to room temperature, filtered and placed in an open container. The use of bulky TBA was dismissed due to the low solubility of its POM salts in water.

$[(CH_3)_4N]_2[H_2V_7O_{21}(H_2O)((CH_2)_3CCH_2OH)]\cdot 4H_2O$ (TMA-1). Orange prismatic single crystals were obtained after ten days using *H_3trisOH* and TMACl. Yield: 153 mg (25 % based on V). Anal. Calcd (found) for $C_{13}H_{45}N_2O_{27}V_7$: C, 15.32 (15.02); H, 4.42 (4.3); N, 2.75 (2.65). FT-IR (KBr): ν (cm^{-1}) = 1485 (f), 1450 (d), 1381 (d), 1284 (d), 1234 (d), 1188 (d), 1165 (f), 1114 (f), 1057 (m), 1026 (f), 987 (f), 956 (F), 914 (f), 810 (f), 790 (f), 729 (f), 636 (m), 594 (f), 532 (f), 497 (f), 424 (f). ^{1}H-RMN (500 MHz, D_2O): δ = 3.24 (s, 24H, $(—N—\mathbf{CH_3})_{TMA}$), 3.50 (s, 2H, $—\mathbf{CH_{2b}}—$), 5.11 (s, 6H, $—\mathbf{CH_{2a}}—$). ^{13}C-RMN (125.7 MHz, D_2O): δ = 40.58 (s, $—CH_{2b}—\mathbf{C}—(CH_{2a})_3$), 55.35 (s, $(—N—\mathbf{C}H_3)_{TMA}$), 60.90 (s, $—CH_{2b}—\mathbf{C}—(CH_{2a})_3$), 76.99 (s, $—\mathbf{C}H_2— O\mu_3$), 88.58 (s, $—\mathbf{C}H_2—O\mu_2$).

$[(CH_3CH_2)_4N]_{1.5}[H_{2.5}V_7O_{21}(H_2O)((CH_2)_3CCH_2OH)]\cdot 4H_2O$ (TEA-2). Orange prismatic single crystals were obtained after nine days using *H_3trisOH* and TEACl. Yield: 132 mg (42 % based on V). Anal. Calcd (found) for $C_{17}H_{49.5}N_{1.5}O_{27}V_7$: C, 19.47 (19.65); H, 4.72 (4.84); N, 2.00 (2.11). FT-IR (KBr): ν (cm^{-1}) = 1485 (f), 1454 (f), 1392 (f), 1304 (d), 1238 (d), 1184 (f), 1119 (f), 1061 (f), 1030 (f), 991 (f), 964 (F), 945 (F), 814 (F), 790 (f), 732 (f), 656 (f), 594 (f), 528 (f), 505 (f), 459 (f), 417 (f).

$[H_3N(CH_2)_2NH_3]_3[HV_7O_{21}(H_2O)((CH_2)_3CCH_2OH)]_2\cdot 2.7H_2O$ (EDA-3). Orange prismatic single crystals were obtained after seven days using *H_3trisOH* and $EDACl_2$. Yield: 140 mg (51 % based on V). Anal. Calcd (found) for $C_{16}H_{59.4}N_6O_{48.7}V_{14}$: C, 10.50 (10.65); H, 3.25 (3.18); N, 4.59 (4.45). FT-IR (KBr): ν (cm^{-1}) = 1520 (d), 1497 (d), 1466 (d), 1381 (d), 1331 (d), 1304 (d), 1242 (d), 1192 (d), 1169 (d), 1120 (f), 1064 (f), 988 (f), 957 (F), 922 (f), 895 (f), 810 (f), 799 (f), 729 (f), 698 (f), 590 (F), 493 (d), 417 (f).

$[(CH_3)_4N]_2[H_2V_7O_{21}(H_2O)((CH_2)_3CCH_3)]\cdot 5H_2O$ (TMA-4). Orange prismatic single crystals were obtained after one week using *H_3trisMe* and TMACl. Yield: 122 mg (20 % based on V). Anal. Calcd (found) for $C_{13}H_{47}N_2O_{27}V_7$: C, 15.29 (15.43); H, 4.61 (4.54); N, 2.75 (2.81). FT-IR (KBr): ν (cm^{-1}) = 1487 (f), 1450 (d), 1396 (d), 1288 (d), 1209 (d), 1126 (m), 1038 (f), 991 (f), 952 (F), 924 (f), 806 (f), 729 (f), 669 (f), 611 (f), 590 (f), 499 (f), 474 (f), 420 (f). ^{1}H-RMN (500 MHz, D_2O): δ = 0.89 (s, 3H, $—\mathbf{CH_3}$), 3.25 (s, 24H, $(-N—\mathbf{CH_3})_{TMA}$), 4.96 (s, 6H, $—\mathbf{CH_2}—$). ^{13}C-RMN (125.7 MHz, D_2O):

δ = 14.50 (s, —C—**C**H_3), 36.25 (s, —$(CH_2)_3$—**C**—CH_3), 55.35 (s, (—N—**C**$H_3)_{TMA}$), 80.54 (s, —**C**H_2—Oμ_3), 88.58 (s, —**C**H_2—Oμ_2).

$[(CH_3)_4N]_2[V_6O_{19}\{(OCH_2)_3CCH_2OH)\}_2]$ (TMA-5). The synthesis of **TMA-5** was similar to that described for **TMA-1** but setting the pH to either 3 or 4, instead of 2. Orange prismatic single crystals were obtained after ten days. Yield: 292 mg (45 % based on V). Anal. Calcd (found) for $C_{18}H_{42}N_2O_{21}V_6$: C, 23.27 (23.18); H, 4.52 (4.51); N, 3.02 (3.10). FT-IR (KBr): ν (cm^{-1}) = 1485 (f), 1450 (d), 1381 (d), 1284 (d), 1238 (d), 1188 (d), 1118 (f), 1060 (f), 1026 (F), 983 (m), 964 (F), 949 (F), 891 (d), 806 (f), 794 (f), 713 (F), 660 (m), 583 (f), 517 (d), 494 (d), 459 (d), 424 (f).

Thermal Analyses. Thermogravimetric and differential thermal analyses (TGA/DTA) were carried out from room temperature to 550 °C at a rate of 5 °C min^{-1} on a TGA/SDTA851e thermobalance under a 50 cm^3 min^{-1} flow of synthetic air (Figure S2 and Table S2). Thermal decomposition of **TMA-1**, **TEA-2**, **EDA-3** and **TMA-4** occurs via two well-differentiated stages and starts at room temperature with a mass loss stage of endothermic nature that corresponds to a dehydration process. Afterwards, the combustion of trisalkoxide ligands and alkylammonium cations takes place and it is accompanied by the breakdown of the POM framework. Both processes develop via several overlapped exothermic events. In the case of anhydrous **TMA-5**, its TGA/DTA curve only displays the later mass loss stage. The obtained final residues correspond in all cases to orthorhombic P*mn*2_1 V_2O_5 as verified by PXRD (PDF: 01-076-1803. Ketelaar, J. A. A. *Chem. Weekbl.* **1936**, *33*, 51−57).

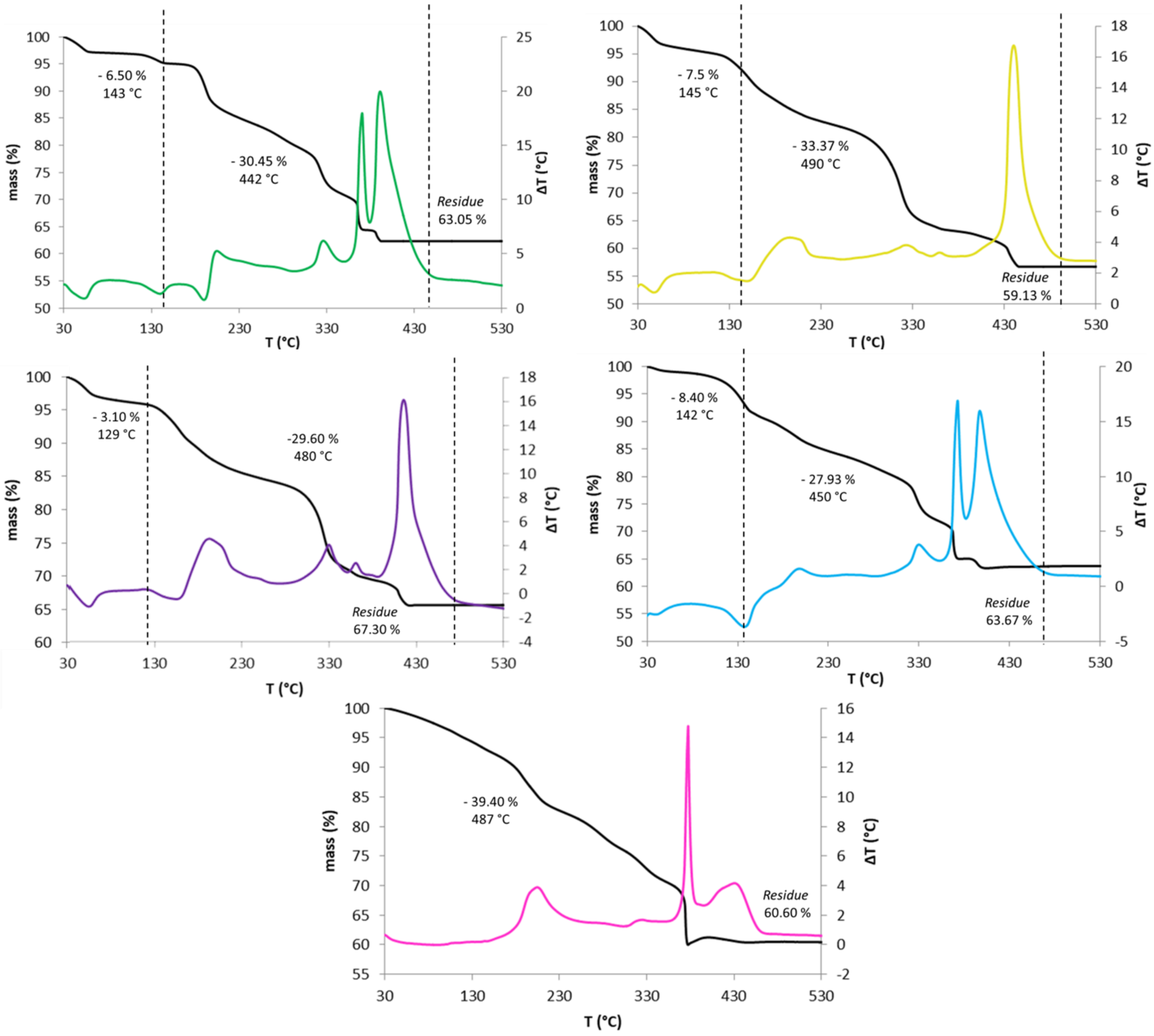

Figure S2. TGA/DTA curves for compounds **TMA-1**, **TEA-2**, **EDA-3**, **TMA-4** and **TMA-5**.

Table S2. Experimental values of the thermogravimetric analyses of **TMA-1**, **TEA-2**, **EDA-3**, **TMA-4** and **TMA-5**, compared to calculated values for each mass loss step.

Compound	Step	T_0 (°C)	T_f (°C)	% $mass_{exp.}$	$\%m(res.)_{exp}$	% $mass_{calc.}$	$\%m(res.)_{calc}$
**TMA-1**	1	30	143	6.50		7.00 (4 H_2O)	
	2	143	442	30.45		30.48	
	Residue				63.05		62.52 (3.5 V_2O_5)
**TEA-2**	1	30	145	7.50		6.87 (4 H_2O)	
	2	145	490	33.37		33.41	
	Residue				59.13		59.72 (3.5 V_2O_5)
**EDA-3**	1	30	129	3.10		2.90 (2.7 H_2O)	
	2	129	480	29.60		28.85	
	Residue				67.30		68.25 (7 V_2O_5)
**TMA-4**	1	30	142	8.40		8.80 (5 H_2O)	
	2	142	450	27.93		28.80	
	Residue				63.67		62.40 (3.5 V_2O_5)
**TMA-5**	1	30	487	39.40		39.05	
	Residue				60.60		60.95 (3 V_2O_5)

Abbreviations: T_0= initial temperature; T_f= final temperature; % $mass_{exp.}$ = experimental mass loss; % $mass_{calc.}$= calculated mass loss; $\%m(res.)_{exp}$ = experimental mass corresponding to the final residue; $\%m(res.)_{calc}$ = experimental mass corresponding to the final residue.

Single-Crystal X-Ray Diffraction. Crystallographic data for compounds **TMA-1, TEA-2, EDA-3, TMA-4** and **TMA-5** are provided in Table S3. Intensity data were collected at 100 K on a Rigaku Oxford Diffraction SuperNova diffractometer equipped with monochromated MoKα radiation (λ = 0.71073 Å) and Eos CCD detector, except for **TMA-5**, for which data were collected at 150 K using monochromated CuKα radiation (λ = 1.543 Å) and Atlas CCD detector. Data frames [unit cell determination, multi-scan or analytical absorption correction with face indexing (**TEA-2**), intensity data integration and correction for Lorentz and polarization effects] were performed using the CrysAlis Pro software package (*CrysAlisPro Software System*; Agilent Technologies UK Ltd.: Oxford, UK, 2012). Structures were solved using OLEX2 (Dolomanov, O. V. et al. *J. Appl. Crystallogr*. **2009**, *42*, 339) and refined by full–matrix least–squares with SHELXL–2014/7 (Sheldrick, G. M. *Acta Crystallogr*. **2008**, *A64*, 112) integrated in WinGX (Farrugia, L. J. *J. Appl. Crystallogr*. **1999**, *32*, 837). Final geometrical calculations were carried out with PLATON (Spek, A. L. *Acta Crystallogr.* **2009**, *D65*, 148) and their visualization was performed using Crystal Maker 10. Bond Valence Sum calculations were carried out using the Brown & Wu method as integrated in Vesta (Brown, I. D.; Wu, K. K. *Acta Crystallogr.* **1976**, *B32*, 1957; Momma, K.; Izumi, F. *J. Appl. Crystallogr.* **2011**, *44*, 1272).

Thermal vibrations were treated anisotropically for all atoms except hydrogen, and ellipsoids of quaternary C atoms and one of the N atoms of **TEA-2** were restrained using standard ISOR commands from SHELX. The hydroxyl group belonging to the *trisOH* moiety in **TMA-1** was disordered over two crystallographic positions showing 80/20 % occupancies. **TMA-5** exhibits a similar disorder (50/50 %), but originating from the tetragonal and highly symmetric $P4_2/m$ space group. One of the EDA cations in **EDA-3** is also disordered over two crystallographic positions with similar population factors. The number of positions suitable for water molecules of hydration that were located in the Fourier maps were 8, 7 and 3 per asymmetric unit of **TMA-1**, **TEA-2** and **EDA-3**, respectively. The population factors of such positions were initially refined without restrictions and rounded to their first decimal in the final refinement cycle. This resulted in a total number of 4 water molecules for **TMA-1** and **TEA-2** and 2.7 water molecules for **EDA-3**, in good agreement with the results obtained from thermogravimetric and elemental analyses. In contrast, 5 water molecules of hydration showing full occupancy were found in **TMA-4**. Furthermore, the good quality of the data allowed us to locate all of the H atoms belonging to the water molecules in the Fourier map of **EDA-3** and **TMA-4**, and they were restrained to their parent sites using DFIX instructions. For the remaining compounds, no H atom was placed over disordered water molecules of hydration due to the poorer quality of the data. Hydrogen atoms of the organic moieties were placed in calculated positions and refined using a riding model with standard SHELXL parameters, whereas protonation sites in POM oxygen atoms (including the O1W coordination water molecule) were located in

Fourier maps and restrained using DFIX commands. Compound **EDA-3** crystallizes in the chiral $P2_12_12_1$ orthorhombic space group. The refinement of this structure yielded a Flack parameter of 0.23(4), and therefore, it was refined considering a racemic, two-component inversion twin.

Table S3. Crystallographic Data for **TMA-1, TEA-2, EDA-3, TMA-4** and **TMA-5.**

	TMA-1	TEA-2	EDA-3*	TMA-4	TMA-5
**formula**	$C_{13}H_{45}N_2O_{27}V_7$	$C_{17}H_{49.5}N_{1.5}O_{27}V_7$	$C_{16}H_{59.4}N_6O_{48.7}V_{14}$	$C_{13}H_{47}N_2O_{27}V_7$	$C_{18}H_{42}N_2O_{21}V_6$
**fw (g mol^{-1})**	1018.09	1047.63	1827.60	1020.11	928.18
**crystal system**	Triclinic	Monoclinic	Orthorhombic	Monoclinic	Tetragonal
**space group**	*P*-1	*C*2/*c*	$P2_12_12_1$	$P2_1/c$	$P4_2/m$
a **(Å)**	9.3593(10)	26.8908(9)	17.1424(4)	14.38651(16)	12.6836(3)
b **(Å)**	12.1910(9)	13.3647(6)	17.3401(4)	21.1713(2)	12.6836(3)
c **(Å)**	17.3023(18)	20.2214(9)	17.3495(4)	11.9874(15)	10.0171(4)
α **(°)**	98.593(7)	90	90	90	90
β **(°)**	103.601(9)	90.144(4)	90	107.7444(13)	90
γ **(°)**	109.184(8)	90	90	90	90
V **(Å^3)**	1756.1(3)	7267.3(5)	5157.1(2)	3477.44(7)	1611.47(11)
**Z**	2	8	4	4	2
ρ_{calcd} **(g cm^{-3})**	1.925	1.838	2.360	1.948	1.913
μ **(mm^{-1})**	1.878	1.814	2.537	1.897	14.816
**reflections collected**	12606	29318	42089	31166	12791
**unique (R_{int})**	6769 (0.072)	7093 (0.162)	10044 (0.104)	6778 (0.031)	1729 (0.082)
**obs. [$I>2\sigma(I)$]**	3641	3675	8914	6126	1345
**parameters**	505	512	797	505	134
**restraints**	4	28	554	15	2
**$R(F)^a$ [$I>2\sigma(I)$]**	0.067	0.068	0.066	0.025	0.040
**$wR(F^2)^b$ [all]**	0.156	0.154	0.178	0.065	0.100
**GoF**	0.991	1.044	1.078	1.040	1.025

$^aR(F)=\Sigma||F_o-F_c||/\Sigma|F_o|$; $^bwR(F^2)=\{\Sigma[w(F_o^2-F_c^2)^2]/\Sigma[w(F_o^2)^2]\}^{1/2}$ *racemic twin, Flack parameter = 0.23(4)

FT-IR Spectroscopy and PXRD Analyses. All the solids originating from the slow evaporation of the final solutions at room temperature were identified by FT-IR spectroscopy. The similarity of the polyanions in **TMA-1**, **TEA-2**, **EDA-3** and **TMA-4**, as well as their successful organic functionalization, were preliminarily verified by FT-IR spectroscopy (Figures S3 to S5) and the homogeneity of the obtained solid crystalline samples was confirmed using powder X-ray diffraction (Figures S6 and S7).

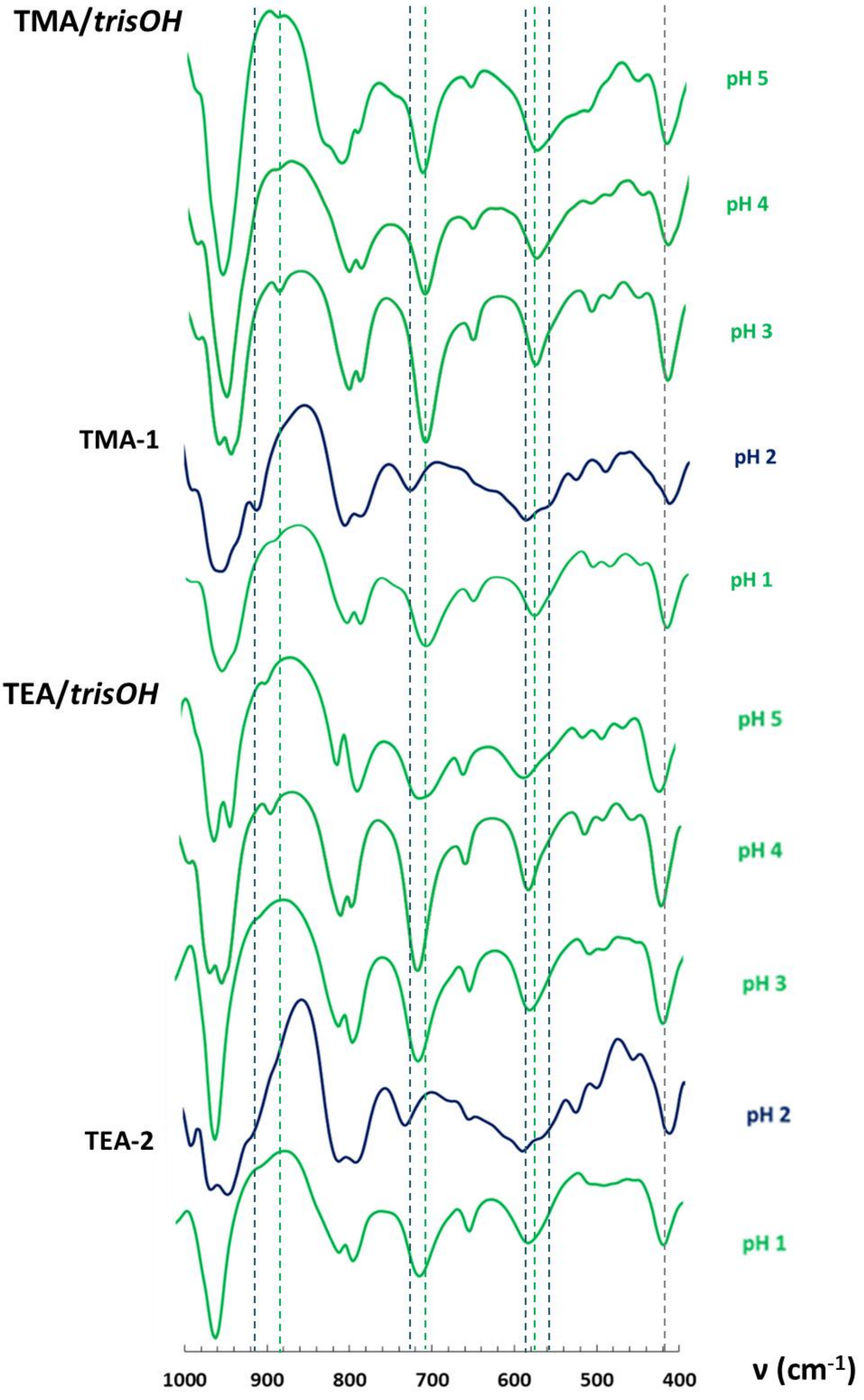

Figure S3. FT-IR spectra in the 1000-400 cm^{-1} region of the solid products obtained from the reaction of $NaVO_3$ with different tripodal ligands and alkylammonium salts in acidic aqueous solution. Color code: green, organically-functionalized hexavanadate; dark blue, organically-functionalized heptavanadate; pink, decavanate; gray, non-identified residue.

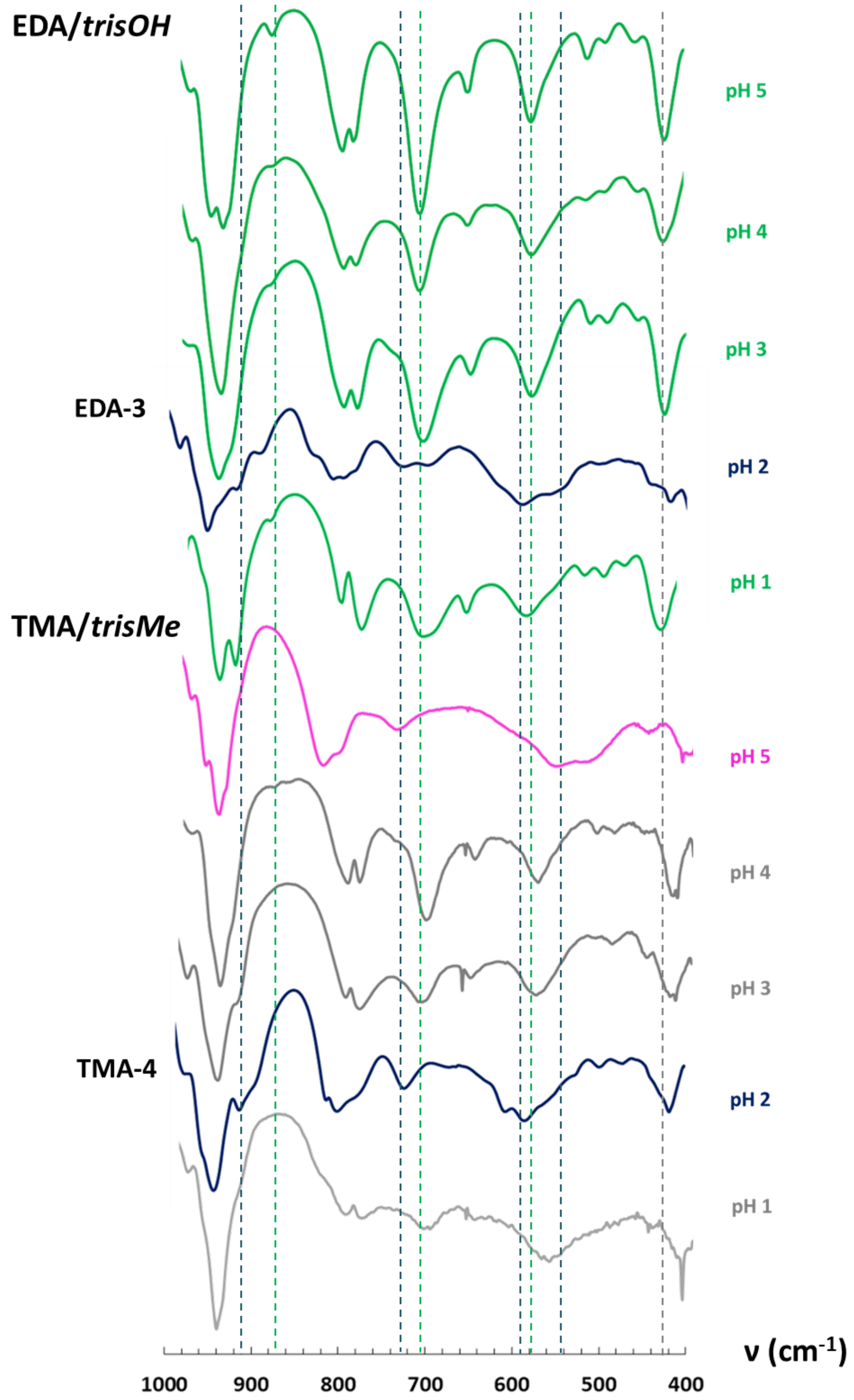

Figure S3 (continued). FT-IR spectra in the 1000-400 cm^{-1} region of the solid products obtained from the reaction of $NaVO_3$ with different tripodal ligands and alkylammonium salts in acidic aqueous solution. Color code: green, organically-functionalized hexavanadate; dark blue, organically-functionalized heptavanadate; pink, decavanate; gray, non-identified residue.

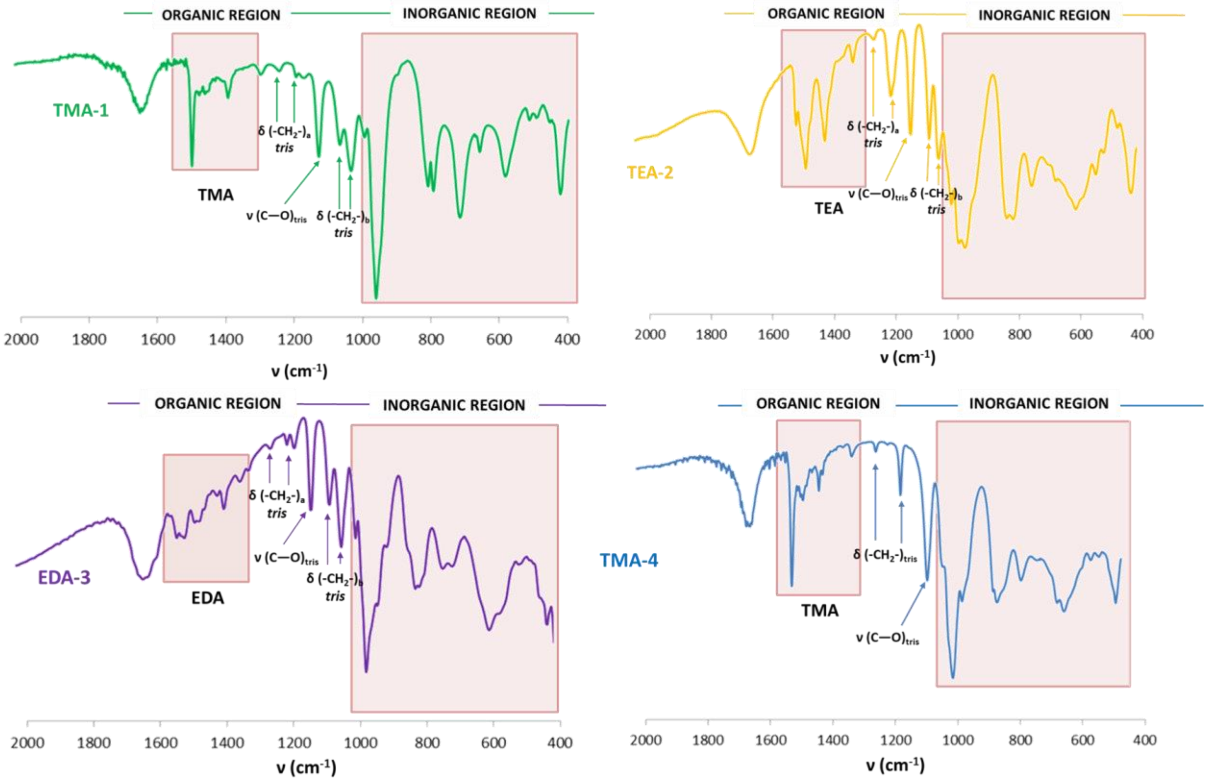

Figure S4. Expanded FT-IR spectra in the 2000 to 400 cm^{-1} region for compounds **TMA-1**, **TEA-2**, **EDA-3** and **TMA-4** (dark blue spectra in Figure S3), together with the assignment of the key signals confirming the successful organic functionalization.

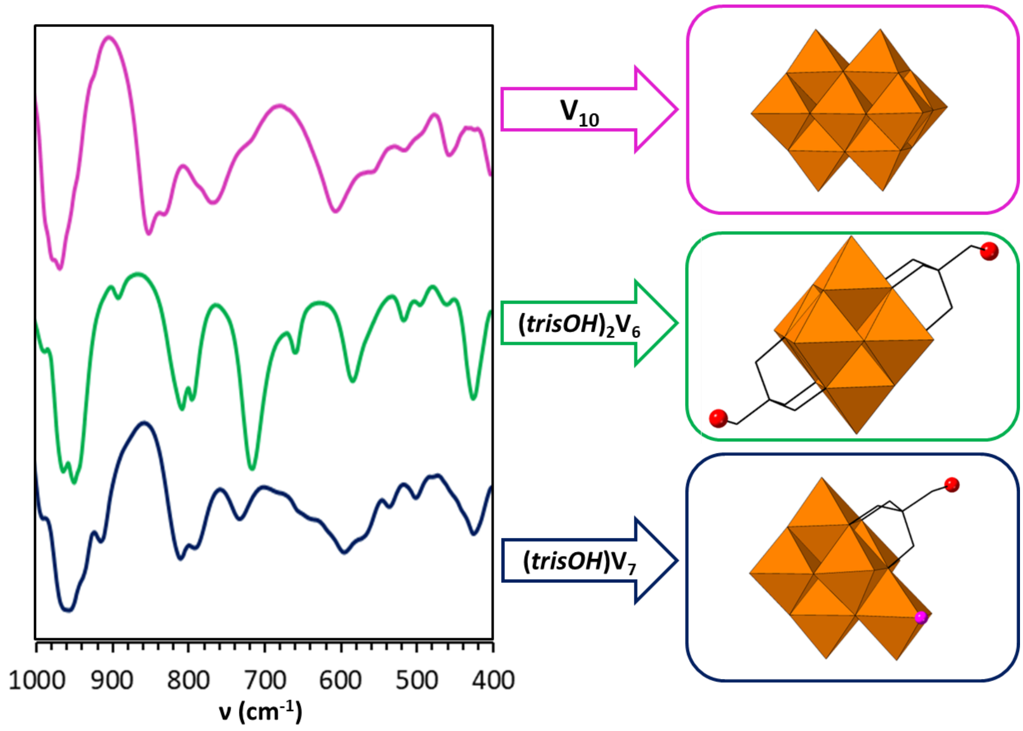

Figure S5. Comparison of the inorganic regions in the FT-IR spectra of $Na_4TMA_2[V_{10}O_{28}]\cdot 20H_2O$ (pink), **TMA-5** (green) and **TMA-1** (dark blue).

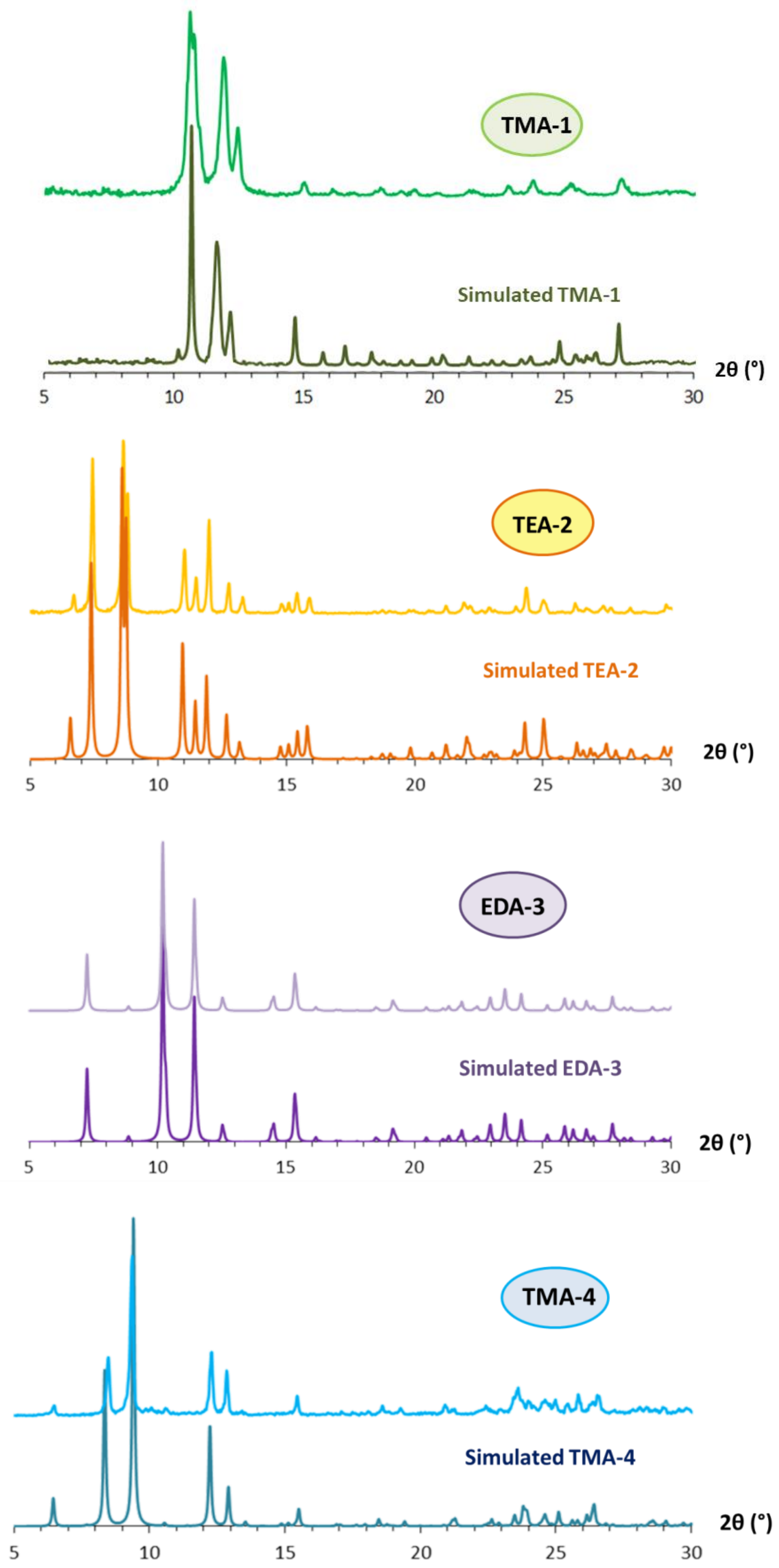

Figure S6. PXRD patterns of compounds **TMA-1**, **TEA-2**, **EDA-3** and **TMA-4** compared with those simulated from their respective single-crystal X-ray diffraction data.

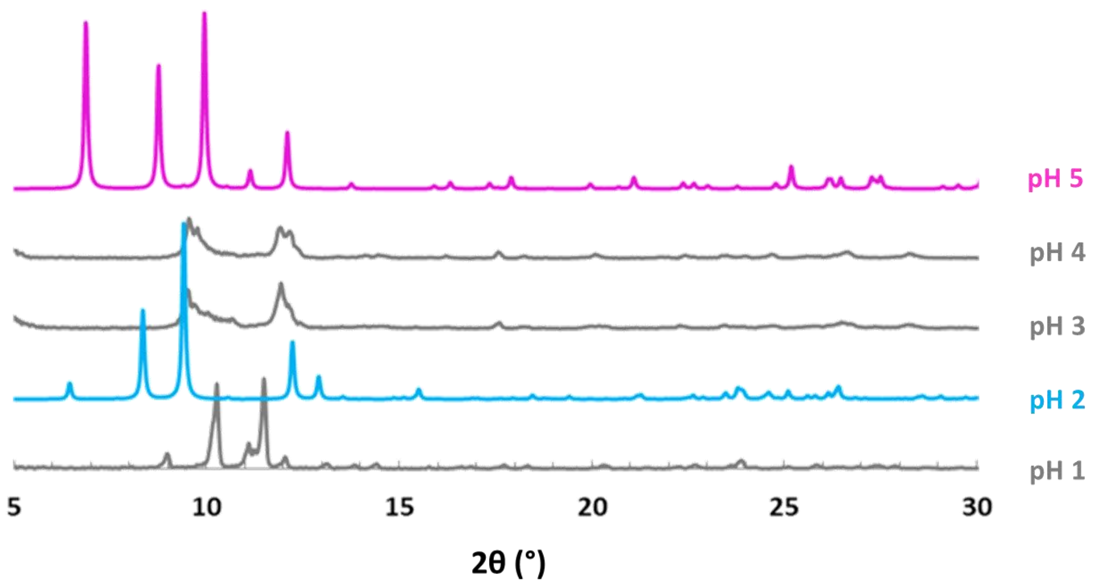

Figure S7. PXRD patterns of the different solid samples obtained from the reaction between $NaVO_3$, the *H_3trisMe* and TMACl at different acidic pH values. Color code: decavanate, pink; non-identified residue, gray; **TMA-4**, blue.

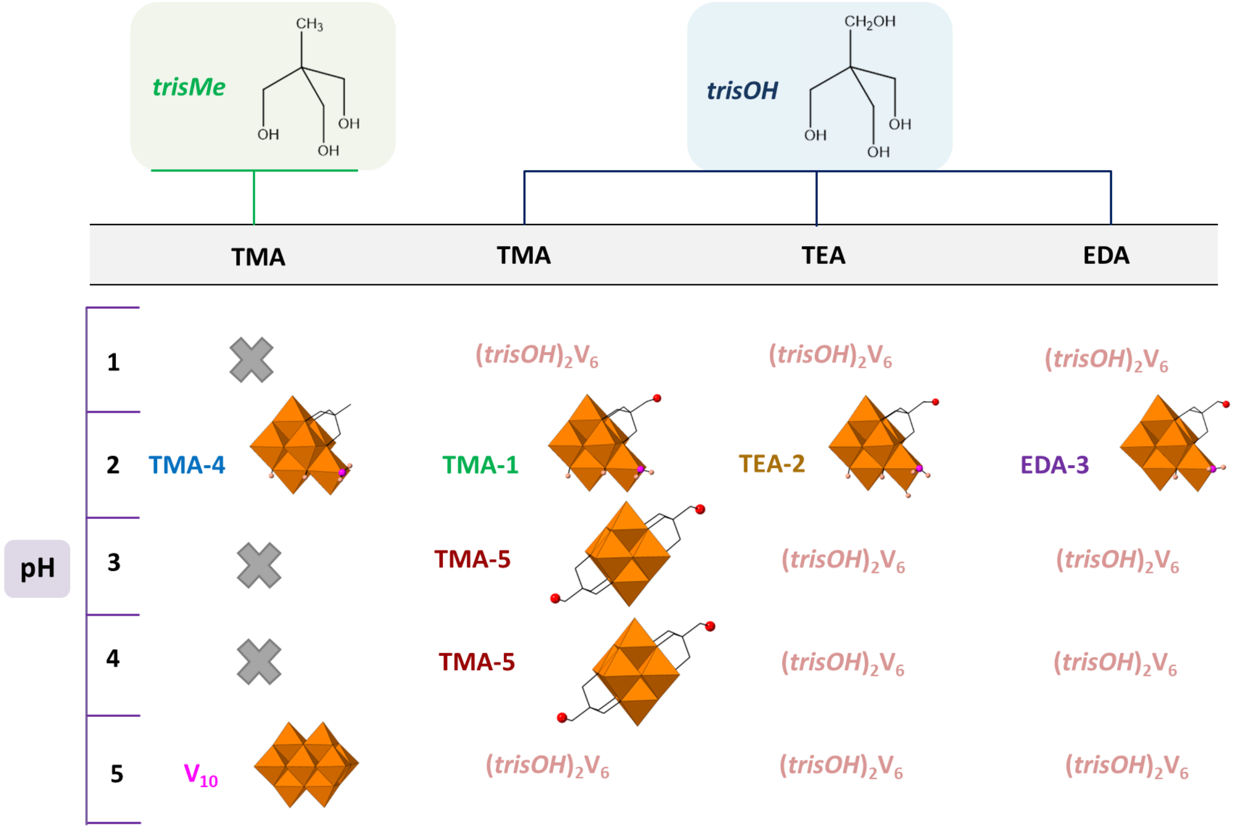

Figure S8. Summary of the results obtained from the reactions between $NaVO_3$, two different tripodal ligands and three different alkylammonium salts as a function of the pH (code: X, non-identified solid residue; (*trisOH*)$_2$V$_6$, samples that display the same hybrid POM as **TMA-5** according to their FT-IR spectra, but could not be analyzed by single-crystal X-ray diffraction).

2.- CRYSTAL STRUCTURE

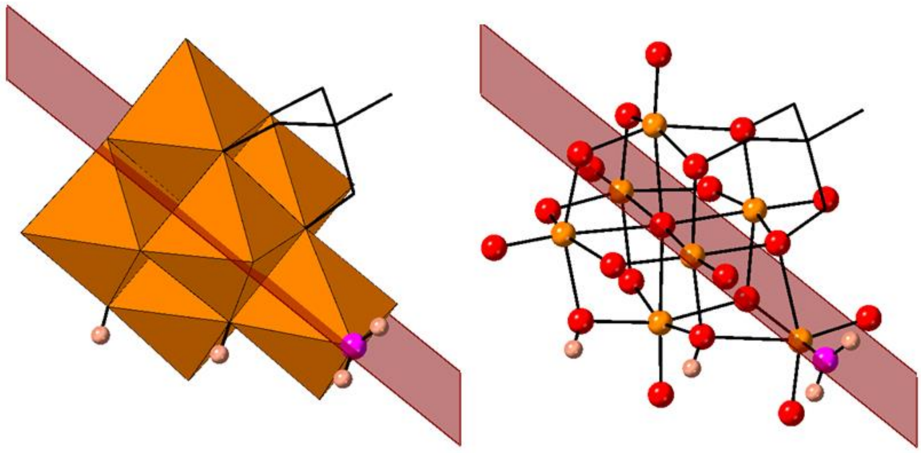

Figure S9. Illustration of the ideal symmetry plane of the inorganic {$V_7O_{22}(H_2O)$} cluster depicted over the crystal structure of **TMA-4** in polyhedral (left) and ball and stick (right) representation.

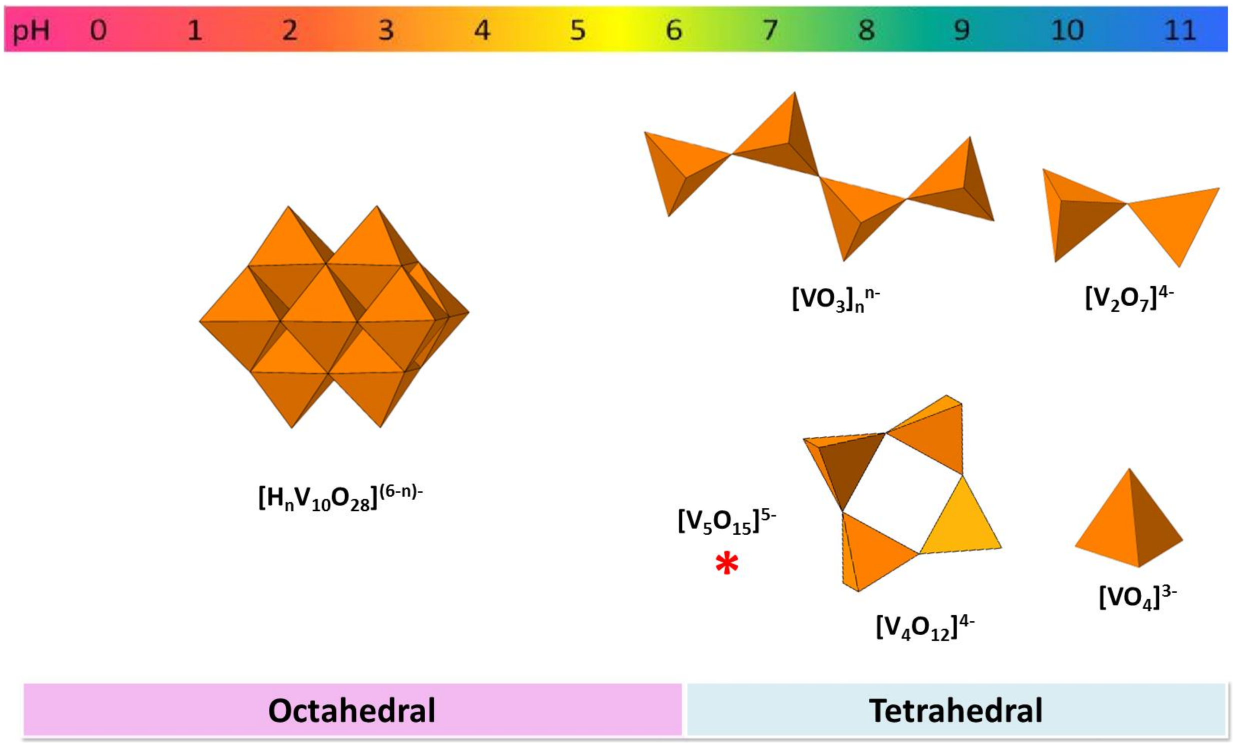

Figure S10. Structure of predominant polyoxovanadate species in aqueous solution as a function of pH. Note that octahedral vanadium centers are observed at acidic pH, whereas species formed at basic pH are constituted by tetrahedral units. The $[V_5O_{15}]^{5-}$ anion (*) has been detected in aqueous solution, but no crystal structure has been reported to date to our knowledge.

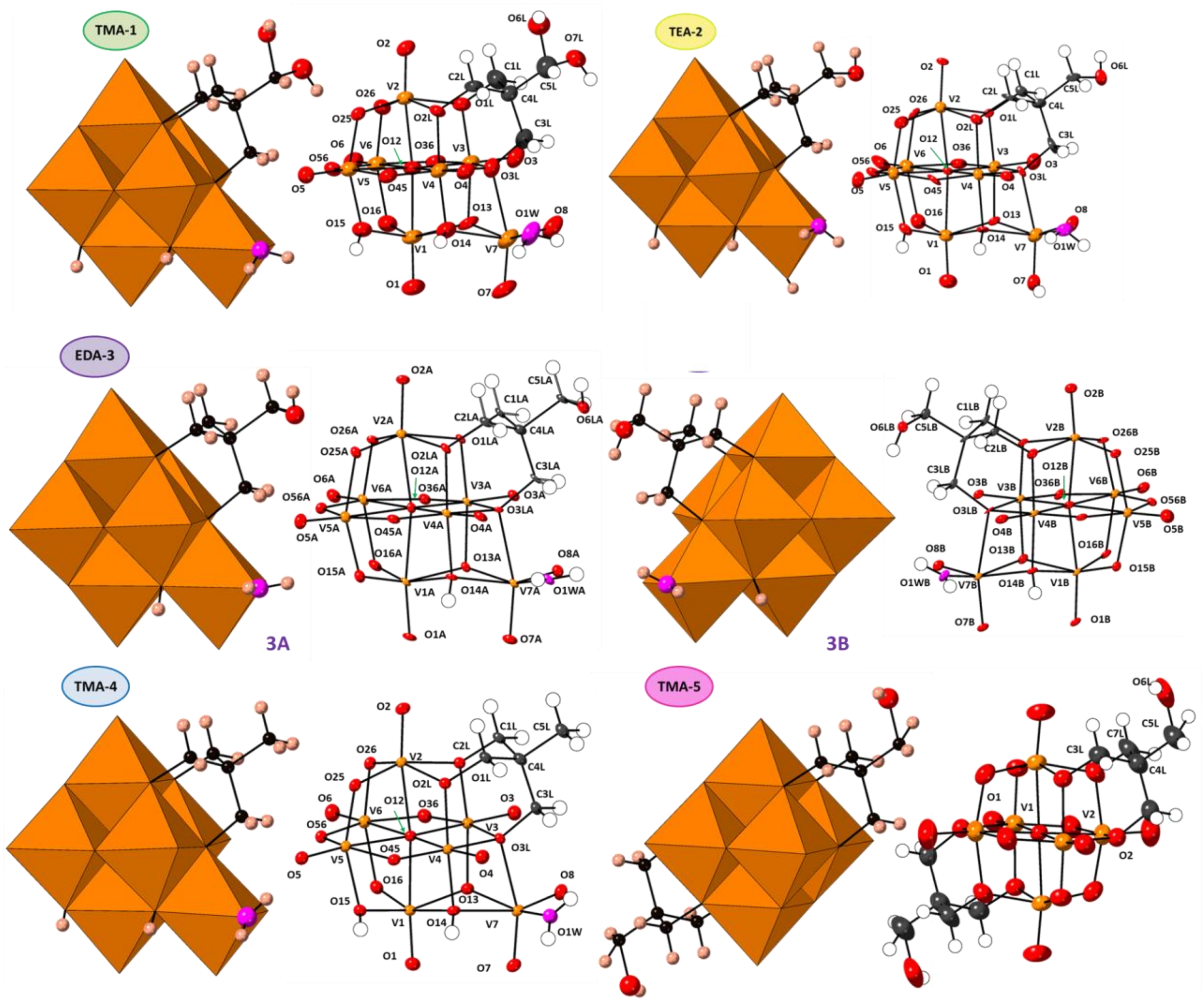

Figure S11. Polyhedral and ORTEP view showing 50% probability ellipsoids of the hybrid heptavanadate units in **TMA-1**, **TEA-2**, **EDA-3**, **TMA-4** and **TMA-5**, together with atom labelling scheme. Color code: V, orange; O, red, C, black; O1W, pink; H, light pink (white in ORTEP representations).

Table S4. V—O bond lengths (Å) in compounds **TMA-1**, **TEA-2**, **EDA-3**, **TMA-4** and **TMA-5**.

	V1	V2	V3	V4	V5	V6	V7
TMA-1	O1 1.598(5)<br>O12 2.031(4)<br>O13 1.845(5)<br>O14 1.784(5)<br>O15 1.993(5)<br>O16 2.252(4)	O2 1.604(5)<br>O2L 2.239(5)<br>O25 1.941(5)<br>O26 2.126(5)<br>O1L 1.944(6)<br>O12 1.730(5)	O3 1.612(5)<br>O3L 2.095(5)<br>O36 1.723(5)<br>O12 2.000(5)<br>O13 1.916(4)<br>O1L 2.164(5)	O4 1.599(5)<br>O3L 2.104(5)<br>O45 1.736(5)<br>O14 2.240(5)<br>O2L 1.941(5)<br>O12 1.959(5)	O5 1.604(5)<br>O56 1.970(5)<br>O12 2.302(4)<br>O45 1.944(5)<br>O15 1.970(5)<br>O25 1.839(5)	O6 1.604(5)<br>O36 1.824(5)<br>O12 2.238(5)<br>O16 2.018(5)<br>O36 1.991(5)<br>O56 1.794(5)	O7 1.621(5)<br>O1W 2.029(5)<br>O8 1.642(5)<br>O14 2.243(5)<br>O13 1.954(4)<br>O3L 2.353(5)
TEA-2	O1 1.597(6)<br>O12 2.287(5)<br>O13 1.929(5)<br>O14 2.158(5)<br>O15 1.933(5)<br>O16 1.724(6)	O2 1.609(5)<br>O2L 2.072(5)<br>O25 1.853(5)<br>O26 1.778(6)<br>O1L 1.948(5)<br>O12 2.232(5)	O3 1.619(5)<br>O3L 2.125(5)<br>O36 1.745(5)<br>O12 2.190(5)<br>O13 1.901(5)<br>O1L 1.948(5)	O4 1.618(5)<br>O3L 2.093(5) .<br>O45 1.72(5)<br>O14 2.017(5)<br>O2L 1.920(5)<br>O12 2.167(5)	O5 1.609(5)<br>O56 1.806(5)<br>O12 2.253(5)<br>O45 2.013(5)<br>O15 2.018(6)<br>O25 1.794(5)	O6 1.590(6)<br>O26 1.898(6)<br>O12 2.336(5)<br>O16 1.948(6)<br>O36 2.004(5)<br>O56 1.823(5)	O7 1.647(6)<br>O1W 2.022(6)<br>O8 1.610(5)<br>O14 2.239(5)<br>O13 1.961(5)<br>O3L 2.305(5)
EDA-3 3A	O1A 1.631(5)<br>O12A 2.222(6)<br>O13A 2.015(5)<br>O14A 2.128(6)<br>O15A 1.824(6)<br>O16A 1.750(6)	O2A 1.614(6)<br>O2LA 2.010(6)<br>O25A 1.754(6)<br>O26A 1.856(6)<br>O1LA 2.093(6)<br>O12A 2.216(6)	O3A 1.616(6)<br>O3LA 2.114(6)<br>O36A 2.177(6)<br>O12A 2.177(6)<br>O13A 1.743(6)<br>O1LA 1.952(5)	O4A 1.612(6)<br>O3LA 2.127(6)<br>O45A 1.718(6)<br>O14A 1.998(5)<br>O2LA 1.931(5)<br>O12A 2.224(5)	O5A 1.606(6)<br>O56A 1.848(6)<br>O12A 2.302(6)<br>O45A 2.011(6)<br>O15A 1.824(6)<br>O25A 1.935(6)	O6A 1.603(6)<br>O26A 1.846(6)<br>O12A 2.301(5)<br>O16A 1.970(6)<br>O36A 1.995(6)<br>O56A 1.815(6)	O7A 1.661(6)<br>O1WA 1.993(6)<br>O8A 1.627(6)<br>O14A 2.238(6)<br>O13A 1.932(6)<br>O3LA 2.268(5)
EDA-3 3B	O1B 1.627(5)<br>O12B 2.218(6)<br>O13B 2.131(6)<br>O14B 2.021(6)<br>O15B 1.842(6)<br>O16B 1.746(6)	O2B 1.613(6)<br>O2LB 2.084(6)<br>O25B 1.847(6)<br>O26B 1.747(6)<br>O1LB 2.015(6)<br>O12B 2.218(6)	O3B 1.706(6)<br>O3LB 2.131(6)<br>O36B 1.706(6)<br>O12B 2.207(6)<br>O13B 1.996(5)<br>O1LB 1.931(6)	O4B 1.613(6)<br>O3LB 2.131(6)<br>O45B 1.731(6)<br>O14B 1.946(6)<br>O2LB 1.941(6)<br>O12B 2.185(6)	O5B 1.598(6)<br>O56B 1.830(6)<br>O12B 2.323(5)<br>O45B 1.991(6)<br>O15B 1.967(5)<br>O25B 1.850(6)	O6B 1.597(6)<br>O26B 1.953(6)<br>O12B 2.283(6)<br>O16B 1.809(6)<br>O36B 2.012(6)<br>O56B 1.844(6)	O7B 1.656(5)<br>O1WB 1.995(6)<br>O8B 1.635(6)<br>O14B 1.927(6)<br>O13B 2.235(5)<br>O3LB 2.237(6)
TMA-4	O1 1.5987(14)<br>O12 2.2899(14)<br>O13 2.1180(15)<br>O14 1.9290(14)<br>O15 1.7280(15)<br>O16 1.9377(15)	O2 1.6160(14)<br>O2L 2.0082(14)<br>O25 1.8115(15)<br>O26 1.8088(14)<br>O1L 2.0454(15)<br>O12 2.2157(14)	O3 1.6013(15)<br>O3L 2.1025(14)<br>O36 1.7318(14)<br>O12 2.1655(14)<br>O13 2.0012(14)<br>O1L 1.9293(14)	O4 1.6059(14)<br>O3L 2.1099(14)<br>O45 1.7420(15)<br>O14 1.9144(14)<br>O2L 1.9457(14)<br>O12 2.2258(14)	O5 1.5953(15)<br>O56 1.8940(15)<br>O12 2.3249(14)<br>O45 1.9182(14)<br>O15 1.9529(15)<br>O25 1.8601(15)	O6 1.6279(14)<br>O26 1.8429(14)<br>O12 2.2137(14)<br>O16 1.9926(15)<br>O36 2.0346(15)<br>O56 1.7736(15)	O7 1.6296(14)<br>O1W 2.0111(15)<br>O8 1.6267(15)<br>O14 1.9527(14)<br>O13 2.2572(14)<br>O3L 2.2852(14)
TMA-5	O12 2.2620(8)<br>O1L 2.011(2)<br>O1L 2.011(2)<br>O1 1.612(3)<br>O21 1.825(3)<br>O21 1.825(3)	O1L 2.026(2)<br>O2 1.600(3)<br>O22 1.8224(19)<br>O2L 2.0010(19)<br>O21 1.831(3)<br>O12 2.2217(5)	—	—	—	—	—

Table S5. BVS values in **TMA-1**, **TEA-2**, **EDA-3** and **TMA-4** (protonated positions highlighted in bold).

	BVS (O7)	BVS (O14)	BVS (O15)	BVS (O1W)
**TMA-1**	1.617	**1.296**	**1.271**	**0.550**
**TEA-2**	**1.426**	**1.250**	**1.220**	**0.550**
**EDA-3**	1.445 (A)<br>1.463 (B)	**1.298 (A)**<br>**1.301 (B)**	1.826 (A)<br>1.868 (B)	**0.593 (A)**<br>**0.583 (B)**
**TMA-4**	1.580	**1.292**	**1.278**	**0.563**

Crystal Packing

Heptavanadate anions showing the highest extent of protonation (**1, 2** and **4**) interact with centrosymmetrically-related analogues through two pairs of strong O_{POM}—H···O_{POM} hydrogen bonds (O14—H14···O45, O15—H15···O4) to arrange in dimers. The O1W coordination water molecule contributes with an additional pair of hydrogen bonds (O1W—H1W···O5) to the stabilization of these dimeric entities in **TMA-1** and **TEA-2**. In both cases**,** dimers are further linked through O_L-H···O_{POM} contacts involving the hydroxyl group of the *trisOH* fragment to form one-dimensional arrangements running parallel to the crystallographic [100] (**TMA-1**) and [110] (**TEA-2**) directions. Hybrid units in **EDA-3** do not interact with each other through protonated faces, but they form supramolecular layers via O6L—H···O_{POM} and O1W—H1W···O_{POM} type connections involving the OH group of the trisalkoxo moiety and the coordination water molecule, respectively. The crystal packing of **TMA-5** can be described as a three-dimensional hydrogen-bonding network that implies the hydroxyl groups of the *trisOH* fragments (Figure S10).

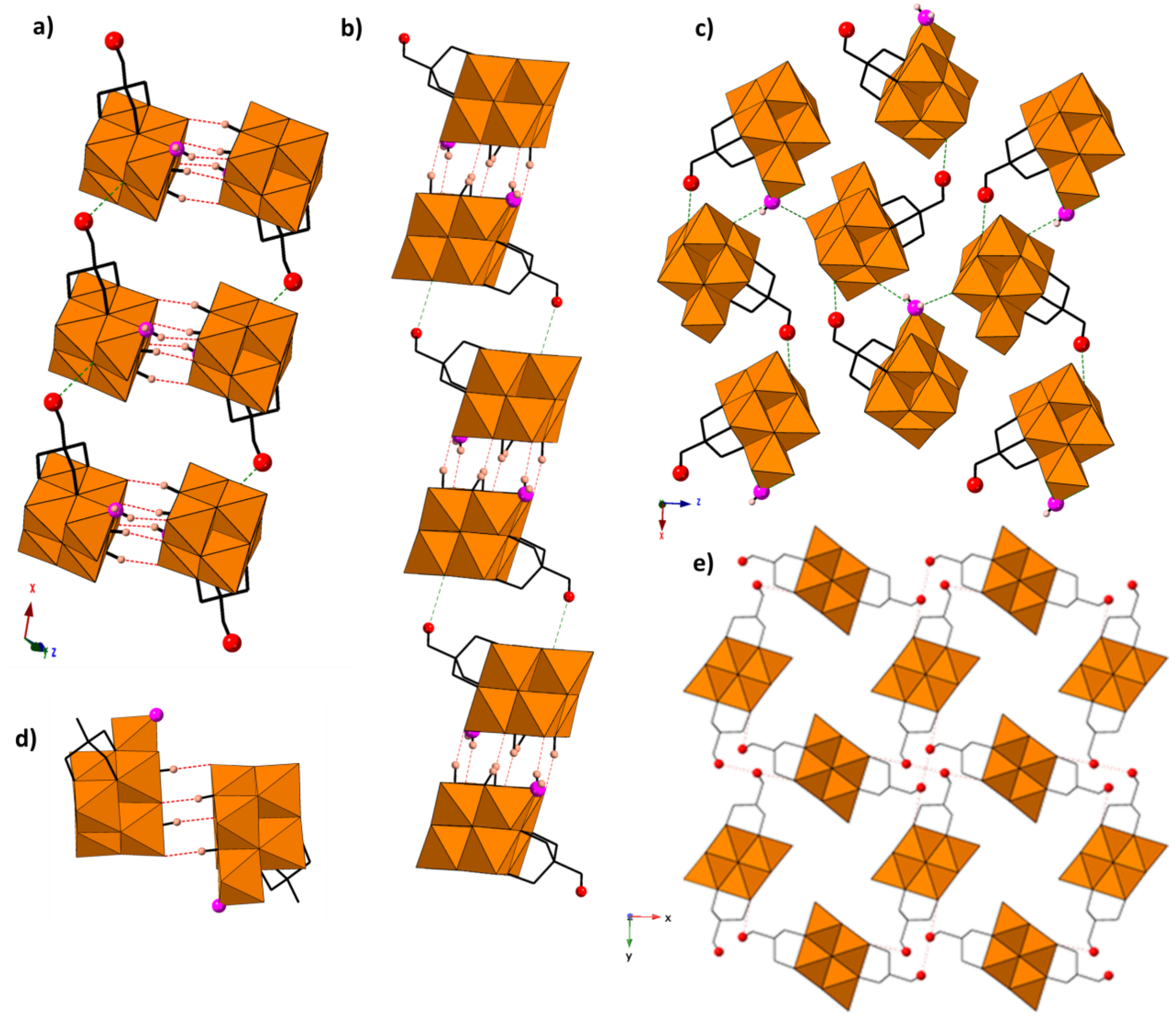

Figure S12. View of the crystal packing of a) **TMA-1**, b) **TEA-2**, c) **EDA-3**, d) **TMA-4** and e) **TMA-5**. Color code: Hydrogen bonds among POMs that form dimeric entities, red dashed lines; other hydrogen bonds, green dashed lines.

Table S6. Donor···Acceptor Distances (Å) for the Hydrogen Bond Interactions in **TMA-1**.

**TMA-1**			
Contacts between POMs			
O14—H14 ···O45^{i}	2.784(7)	O1W—H1WA···O5^{i}	2.706(8)
O15—H15···O4^{ii}	2.712(7)	O6L—H6L···O13^{iii}	2.893(9)
O_W—H···O type contacts			
O1W—H1WB···O3W^{iv}	2.446(13)	O1W—H1WB···O4W^{iv}	2.718(12)
C—H···O type contacts			
C2A—H2AC···O3^{v}	3.200(11)	C5A—H5AA···O26^{vi}	3.425(11)
C3A—H3AA···O2W^{v}	3.25(2)	C3B—H3BB···O6	3.426(9)
C3A—H3AB···O8	3.391(11)	C4B—H4BA···O46	3.446(9)
C3A—H3AC···O2^{vi}	3.263(12)	C5B—H5BA···O25	3.364(9)
C3A—H3AC···O26^{vi}	3.365(13)	C2L—H2LA···O16^{vii}	3.424(9)
C4A—H4AA···O3^{viii}	3.250(12)	C2L—H2LB···O1^{ix}	2.992(9)
C4A—H4AA···O5W^{v}	3.257(19)	C2A—H2AA···O2W^{v}	3.35(2)
C4A—H4AC···O6L^{x}	3.513(12)	C2A—H2AB···O26^{vi}	3.417(11)
C4A—H4AC···O7L^{xi}	3.28(3)	C2A—H2AC···O8W^{vi}	3.27(2)
C5A—H5AB···O7^{vi}	3.321(12)		

Symmetry codes: i) 1-x,2-y,1-z; ii)3/2-x,1/2-y,1-z;iii) 1+x,y,z ; iv) 1+x,1+y,z; v) -x,2-y,-z; vi) x,1+y,z; vii) 1+x,y,z; viii) -x,2-y,-z; ix) 1+x,y,z; x) 1-x,2-y,-z; xi) 1-x,2-y,-z.

Table S7. Donor···Acceptor Distances (Å) for the Hydrogen Bond Interactions in **TEA-2**.

**TEA-2**			
Contacts between POMs			
O14—H14—O45^{i}	2.762(6)	O1W—H···O5^{i}	2.738(7)
O15—H···O4^{i}	2.684(7)	O6L—H···O13^{ii}	2.863(7)
O_W—H···O type contacts			
O1W—H1WA···O7W	2.706(14)	O1W—H1WA···O8W	2.558(13)
C—H···O type contacts			
C1L—H1LB···O3^{ii}	3.006(9)	C8A—H8AA···O15^{iii}	3.239(10)
C4A—H4AA···O2	3.162(10)	C9A—H9AA···O4^{iv}	3.385(10)
C4A—H4AA···O25	3.447(11)	C4B—H4BB···O13^{v}	3.395(10)
C6A—H6AB···O7^{i}	3.425(10)		

Symmetry codes: i) 3/2-x,1/2-y,1-z; ii) 1-x,-y,1-z; iii) x,-y,-1/2+z; iv) 3/2-x,-1/2+y,1/2-z; v) 1-x,1-y,1-z.

Table S8. Donor···Acceptor Distances (Å) for the Hydrogen Bond Interactions in **EDA-3**.

**EDA-3**			
Contacts between POMs			
O1WA—H1WB···O1B^{i}	2.684(8)	O1WA—H1WA·· O26B^{ii}	2.609(9)
O1WB—H1WD·· O1A^{iii}	2.662(8)	O1WB—H1WD·· O2A^{ii}	3.043(7)
O1WB—H1WC·· O26B^{ii}	2.598(9)	O6LA—H6LA·· O56B^{ii}	2.705(7)
O6LB—H6LB·· O56A^{ii}	2.744(7)	O14A—H14A·· O7B^{i}	2.727(7)
O14B—H14B·· O7A^{iii}	2.727(8)		
O_W—H···O type contacts			
O2W—H2WA·· O36B	2.702(11)	O2W—H2WB·· O3W	2.741(19)
O4W—H4WA · O25A	2.805(9)	O4W—H4WB·· O25B	2.837(9)
C—H···O type contacts			
C2D—H2DA·· O5A	3.010(19)	C3A—H3AA·· O6A^{ii}	3.200(10)
C3B—H3BB·· O1B	3.018(12)	C3D—H3DA·· O8A^{iii}	3.200(19)
C3LA—H3LB·· O1^{ii}	3.321(10)	C3LA—H3LA·· O6B^{ii}	3.378(10)
N—H···O type contacts			
N1A—H1AA·· O5B^{iv}	2.909(9)	N1A—H1AB·· O6LA^{v}	2.749(9)
N1B—H1BB·· O13B	2.806(10)	N1B—H1BA·· O3W	2.945(15)
N1D—H1DC·· O8B^{i}	3.046(14)	N1D—H1DA·· O15A	2.799(14)
N1D—H1DB·· O36A^{vi}	3.007(15)	N1D—H1DB·· O1LA^{vi}	2.889(13)
N1E—H1EC·· O7A^{iii}	2.874(10)	N1E—H1EB·· O7B^{i}	2.872(10)
N1E—H1EA·· O45A	3.045(10)	N1E—H1EA·· O45B	2.861(10)
N4A—H4AA·· O3B	2.898(9)	N4A—H4AA·· O8B	2.898(9)
N4B—H4BB·· O2B^{vii}	2.927(10)	N4B—H4BA·· O4A^{iii}	2.870(9)

Symmetry codes: i) 1-x,1/2+y,1/2-z ; ii) 1/2+x,3/2-y,1-z; iii) 1-x,-1/2+y,1/2-z; iv) 1/2-x,1-y,1/2+z; v) 1-x,-1/2+y,3/2-z; vi) 1/2-x,2-y,-1/2+z; vii) 1/2-x,1-y,-1/2+z.

Table S9. Donor···Acceptor Distances (Å) for the Hydrogen Bond Interactions in **TMA-4.**

**TMA-4**			
Contacts between POMs			
O14—H14—O5^{i}	2.755(2)	O15—H15···O45^{i}	2.691(2)
O_W—H···O type contacts			
O1W—H1WA···O2W	2.633(2)	O1W—H1WB···O3W	2.644(2)
O2W—H2WB·· O5^{i}	2.785(2)	O2W—H2WA···O7^{ii}	2.769(2)
O3W—H3WA···O8^{ii}	2.802(2)	O3W—H3WB···O5W	2.799(3)
O4W—H4WB···O2^{iii}	3.063(3)	O4W—H4WA···O3W	2.929(3)
O5W—H5WB···O10	2.867(3)	O6W—H10B···O3	2.965(2)
O6W—H10B ··O8	3.069(2)	O6W—H10A···O26^{iv}	2.892(2)
C—H···O type contacts			
C2A—H2AC···O1^{i}	3.223(3)	C2A—H2AB···O25	3.436(3)
C2B—H2BB···O7^{v}	3.260(3)	C2L—H2LB···O4W^{iii}	3.493(3)
C3A—H3AC···O25^{v}	3.405(3)	C3B—H3BA···O1	3.199(3)
C3B—H3BA···O16	3.463(3)	C3B—H3BC···O16^{vi}	3.281(3)
C3B—H3BB···O2W^{vi}	3.462(3)	C3L—H3LB···O3W	3.331(3)
C4A—H4AA···O26^{v}	3.360(3)	C4B—H4BA···O4W^{v}	3.402(3)
C4B—H4BC··· O13	3.248(3)	C5A—H5AB···O5	3.297(3)
C5A—H5AA···O7^{i}	3.367(3)	C5A—H5AB···O25	3.439(3)

Symmetry codes: i) -x,1-y,-z; ii) x,3/2-y,-1/2+z; iii) 1-x,1-y,-z; iv) 1-x,1-y,1-z; v) x,1/2-y,-1/2+z; vi) -x,1-y,1-z; vi)x,3/2-y,1/2+z; v) x,y,1+z.

Table S10. Donor···Acceptor Distances (Å) for the Hydrogen Bond Interactions in **TMA-5.**

**TMA-5**			
Contacts between POMs			
O6L—H5LB···O6L^{i}	2.438(10)	O6L—H6L···O2^{ii}	2.766(7)
C—H···O type contacts			
C2—H2B···O22	3.448(5)	C2—H2C···O22^{iii}	3.448(5)
C3—H3B···O21^{ii}	3.419(6)	C5L—H5LA···O2^{iv}	3.125(4)
C5L—H5LA ··O2^{v}	3.125(4)	C7L—H7LA···O6L^{vi}	3.198(7)
C7L—H7LB···O6L^{vi}	3.198(7)		

Symmetry codes: i) x,y,1-z; ii) 1-y,x,3/2-z; iii) 1-x,-y,2-z; iv) 1-y,x,-1/2+z; v) 1-y,x,3/2-z; vi) -1+x,y,1-z; vi) -1+x,y,z.

3.- SOLUTION STABILITY

Table S11. Integration of the ^{1}H-RMN signals for an aqueous solution of **TMA-4** after 0, 1, 7, 14 and 21 days referenced to that of TMA cations (24 H, s, 3.25 ppm) and compared to free H_3*trisMe*.

	**Free ligand**		**Condensed ligand**		**Condensed:free t*risMe***
**Signal**	$-C\mathbf{H_3}$	$-C\mathbf{H_2}OH$	$-C\mathbf{H_3}$	$-C\mathbf{H_2}O_{POM}-$	-
**Mult., δ (ppm)**	s, 0.86	s, 3.50	s, 0.89	s, 4.96	-
**Free ligand**	3	6	-	-	-
$\boldsymbol{t_0}$	-	-	3	6	1:0
**1 days**	0.3	0.5	2.7	5.5	9:1
**7 days**	0.5	1	2.5	5	5:1
**14 days**	0.7	1.5	2.3	4.5	3.3:1
**21 days**	0.7	1.5	2.3	4.5	3.3:1

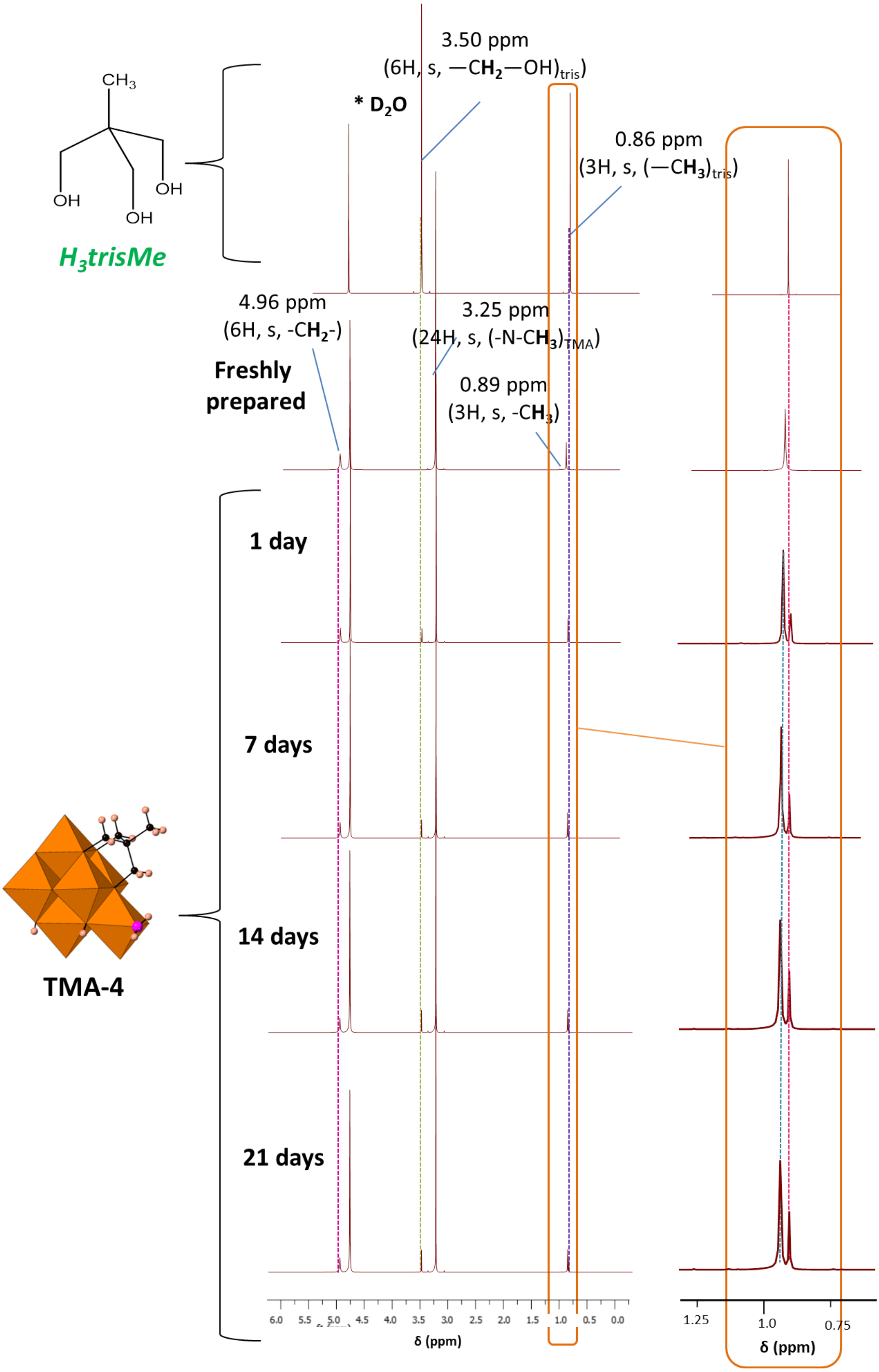

Figure S13. ^{1}H-RMN spectrum of a freshly prepared aqueous solution of **TMA-4** together with those recorded after 1, 7, 14 and 21 days and compared to that of the free *H₃trisMe* ligand provided as reference.

Table S12. ^{13}C-RMN signals of an aqueous solution of **TMA-4** after 0 and 7 days compared to those of H_3*trisMe*.

	**Free ligand**			**Condensed ligand**			
**Signal**	$-CH_3$	$(HOCH_2)_3$-**C**-CH_3	-CH_2-OH	$-CH_3$	$(HOCH_2)_3$-**C**-CH_3	—CH_2—$O_{\mu3}$	—CH_2—$O_{\mu3}$
**δ (ppm)**	15.51	41.07	64.42	14.50	36.25	80.54	88.58
**Free ligand**	✔	✔	✔	X	X	X	X
t₀	X	X	X	✔	✔	✔	✔
**7 days**	✔	✔	✔	✔	✔	✔	✔

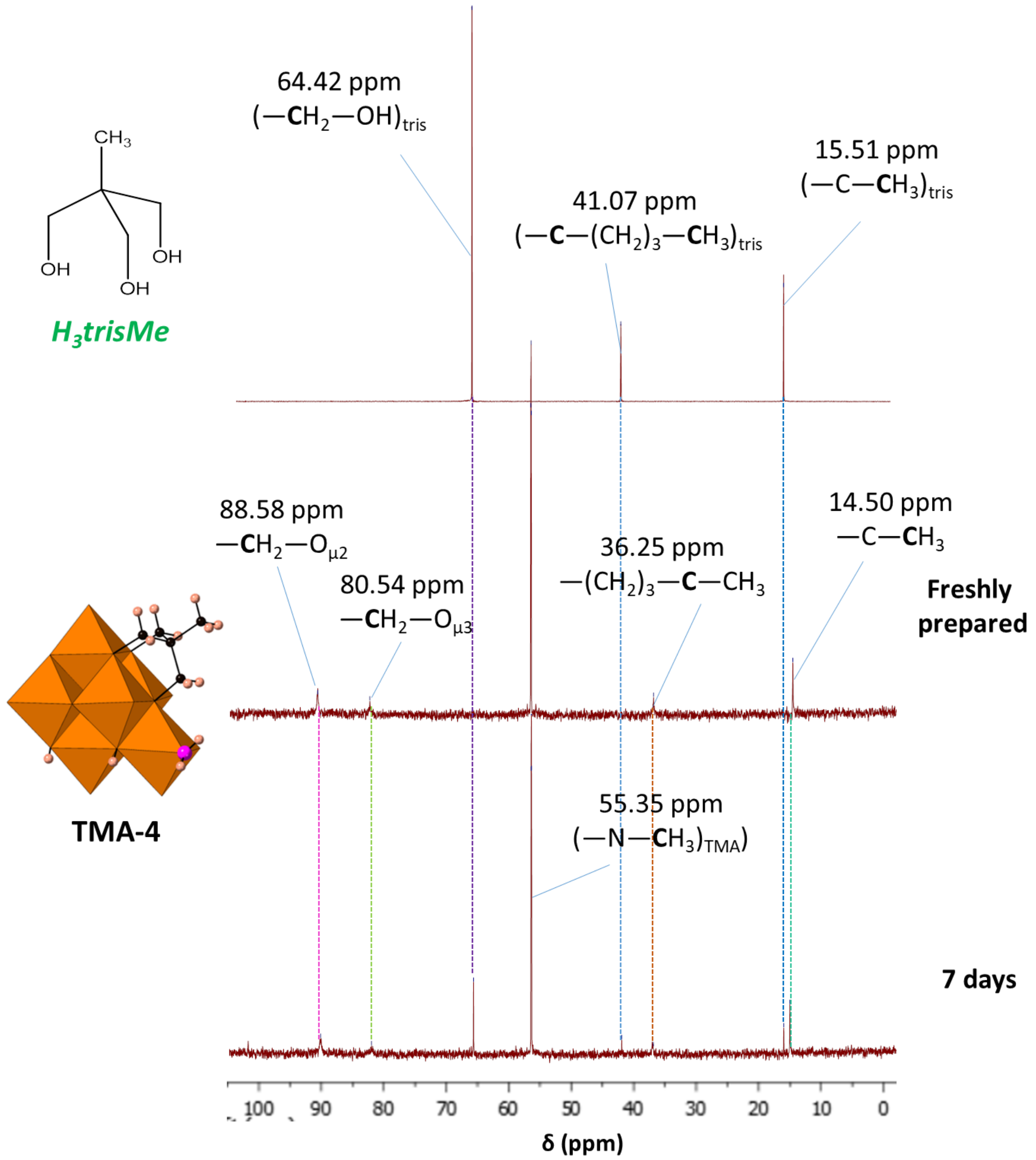

Figure S14. ^{13}C-RMN s spectrum of a freshly prepared aqueous solution of **TMA-4** together with that recorded after 7 days and compared to that of the free H_3*trisMe* ligand provided as reference.

Table S13. Integration of the ^{1}H-RMN signals for an aqueous solution of **TMA-1** after 0, 1, 7, 14 and 21 days referenced to that of TMA cations (24H, s, 3.24 ppm) and compared to free H_3*trisOH*.

	**Free ligand**	**Condensed ligand**		**Condensed:free t*risOH***
**Signal**	–C**H**$_2$–OH	–C**H$_{2b}$**–OH	–C**H$_{2a}$**–O$_{POM}$–	-
**δ (ppm)**	s, 3.50	s, 3.49	s, 5.11	-
**Free ligand**	8	-	-	-
t$_0$	-	2	6	1:0
**1 days**	2.38 (a.d)		5.62	14:1
**7 days**	2.70 (a.d.)		5.30	7:1
**14 days**	3.14 (a.d.)		4.86	4:1
**21 days**	3.12 (a.d.)		4.88	4:1

a.d. = apparent doublet

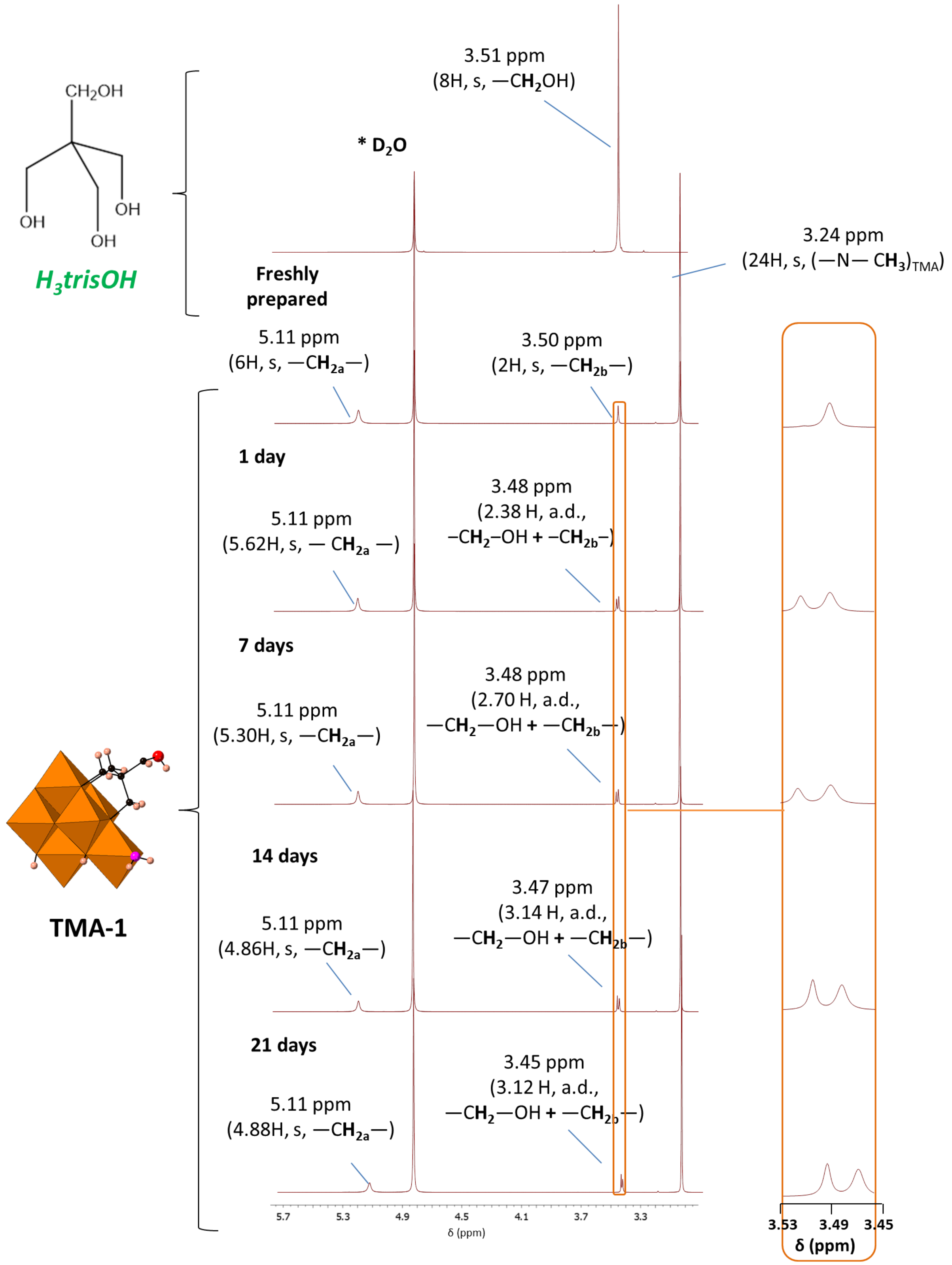

Figure S15. ^{1}H-RMN spectrum of a freshly prepared aqueous solution of **TMA-1** together with those recorded after 1, 7, 14 and 21 days and compared to that of the free *H₃trisOH* ligand provided as reference.

Table S14. ^{13}C-RMN signals of an aqueous solution of **TMA-1** after 0 and 7 days compared to those of H_3*trisOH.*

	**Free ligand**		**Condensed ligand**			
**Signal**	**C**$(CH_2OH)_4$	–**C**H_2–OH	–**C**$(CH_2)_4$–	–**C**H_{2b}–OH	–**C**H_2–$O_{\mu3}$ (POM)	–**C**H_2–$O_{\mu2}$ (POM)
**δ (ppm)**	45.18	61.07	40.58	60.90	76.99	85.89
**Free ligand**	✔	✔	X	X	X	X
t_0	X	X	✔	✔	✔	✔
**7 days**	✔	✔	✔	✔	✔	✔

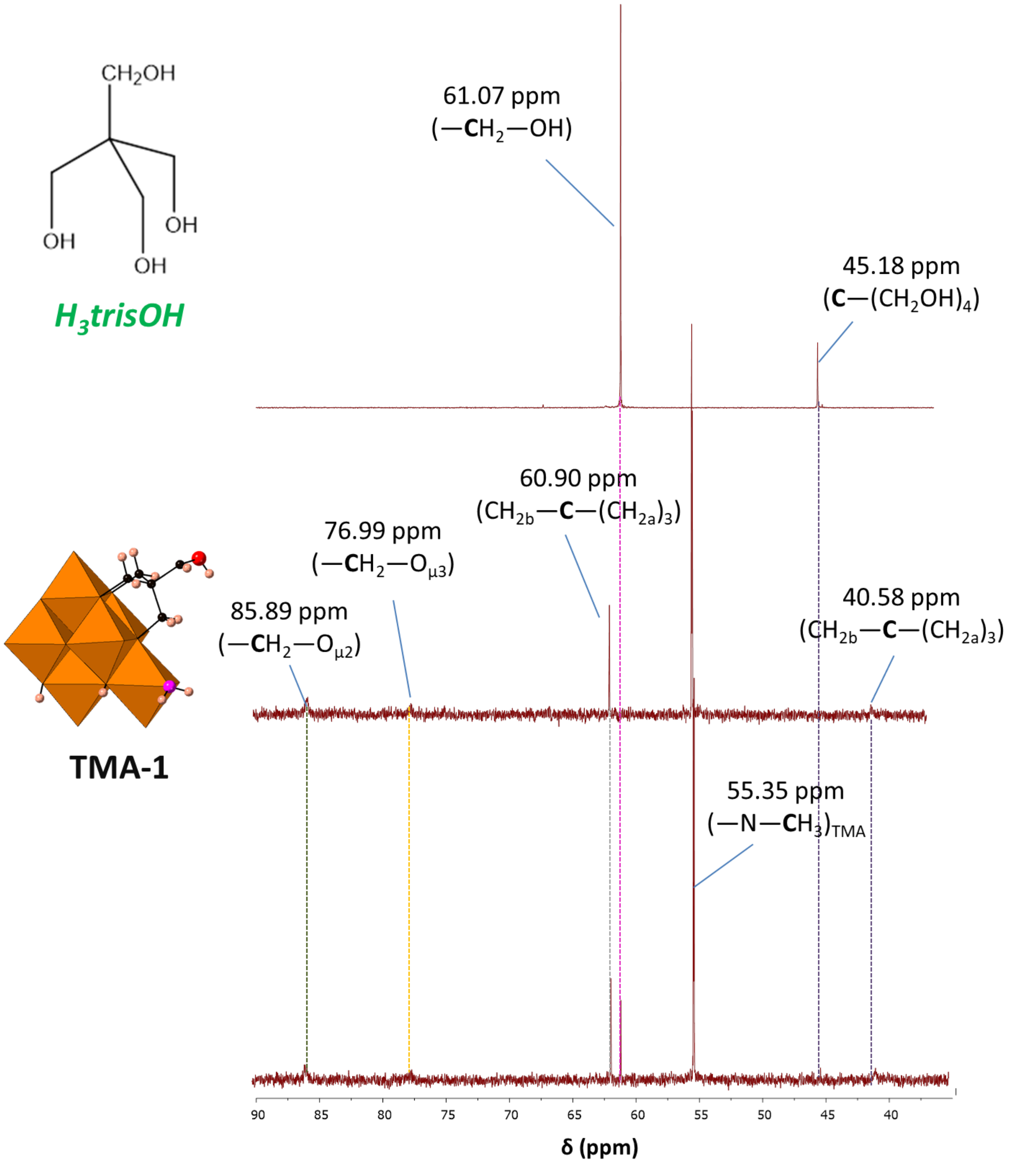

Figure S16. ^{13}C-RMN spectrum of a freshly prepared aqueous solution of **TMA-1** together with that recorded after 7 days and compared to that of the free *H_3trisOH* ligand provided as reference.

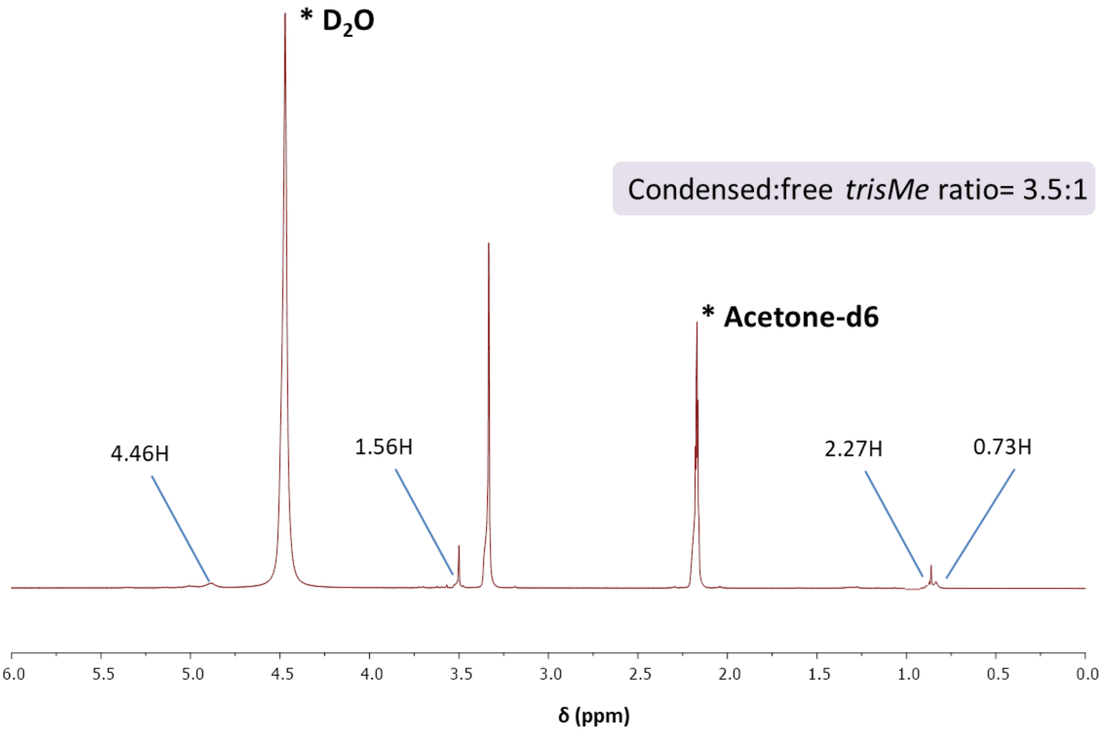

Figure S17. ^{1}H-RMN spectrum recorded after 14 days of a sample of **TMA-4** dissolved in an acetone:water mixture (2:1).

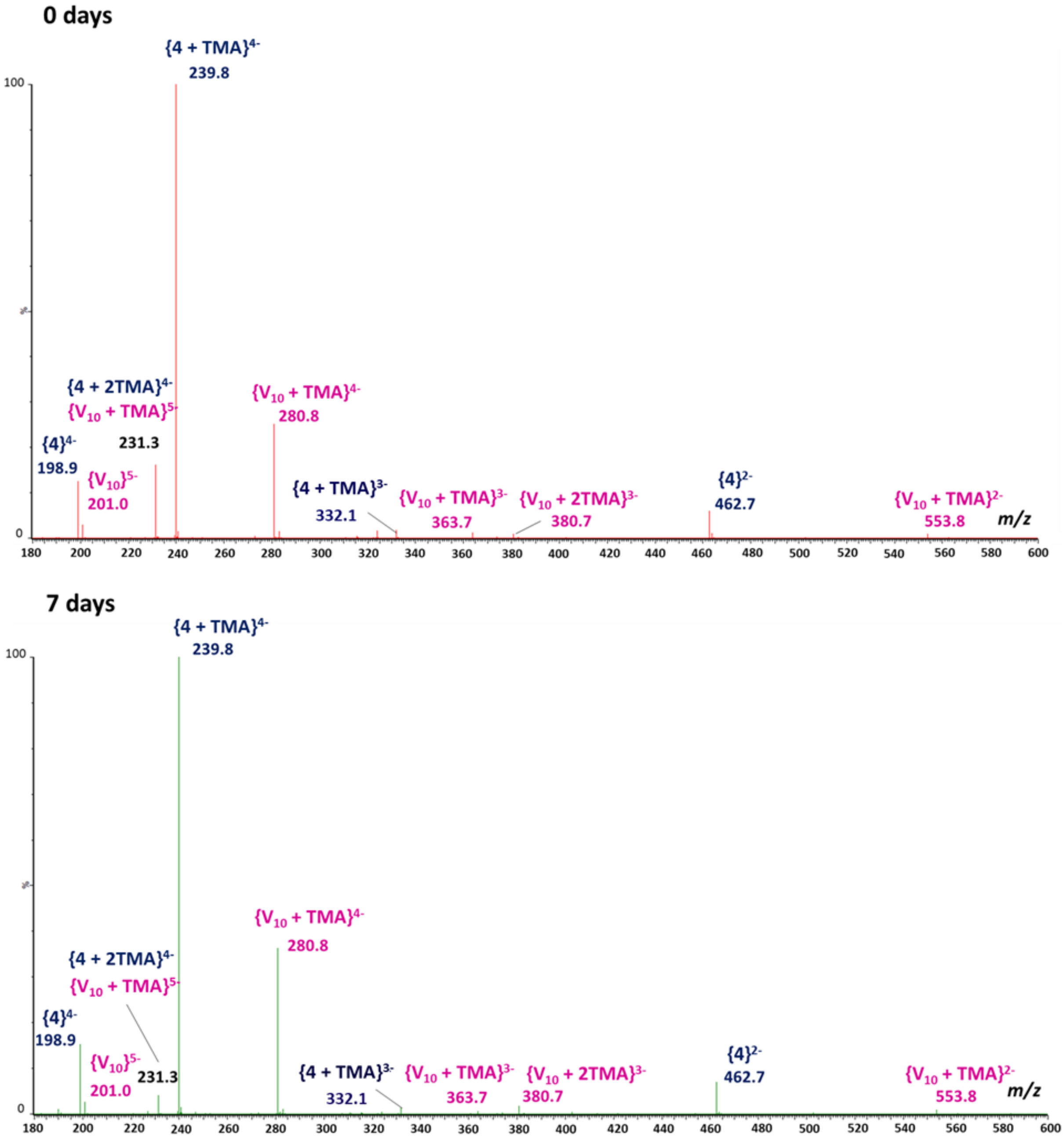

Figure S18. ESI-MS spectra of a freshly prepared sample of **TMA-4** in water:acetonitrile (1:1) and that recorded after one week. Abbreviations: {**4+TMA}$^{4-}$**= [$V_7O_{22}C_5H_9$ + TMA + H + 6H_2O]$^{4-}$; **{4}$^{4-}$**= [$V_7O_{22}C_5H_9$ + 2H + H_2O]$^{4-}$; **{4+TMA}$^{3-}$**= [$V_7O_{22}C_5H_9$ + TMA + 2H + 4H_2O]$^{3-}$; **{4}$^{2-}$=** [$V_7O_{22}C_5H_9$ + 4H + 8H_2O]$^{2-}$; **{V_{10}}$^{5-}$**= [$V_{10}O_{28}$ + H + 3H_2O]$^{5-}$; **{V_{10}+TMA}$^{4-}$**= [$V_{10}O_{28}$ + TMA + H + 5H_2O]$^{4-}$; **{V_{10}+TMA}$^{3-}$**= [$V_{10}O_{28}$ + TMA + 2H + 3H_2O]$^{3-}$; **{V_{10}+2TMA}$^{3-}$**= [$V_{10}O_{28}$ + 2TMA + H + 2H_2O]$^{3-}$; **{V_{10}+TMA}$^{2-}$**= [$V_{10}O_{28}$ + TMA + 3H + 4H_2O]$^{3-}$; **{4+2TMA}$^{4-}$**= [$V_7O_{22}C_5H_9$ + 2TMA]$^{4-}$; **{V_{10}+TMA}$^{5-}$**= [$V_{10}O_{28}$ + TMA + 5H_2O].

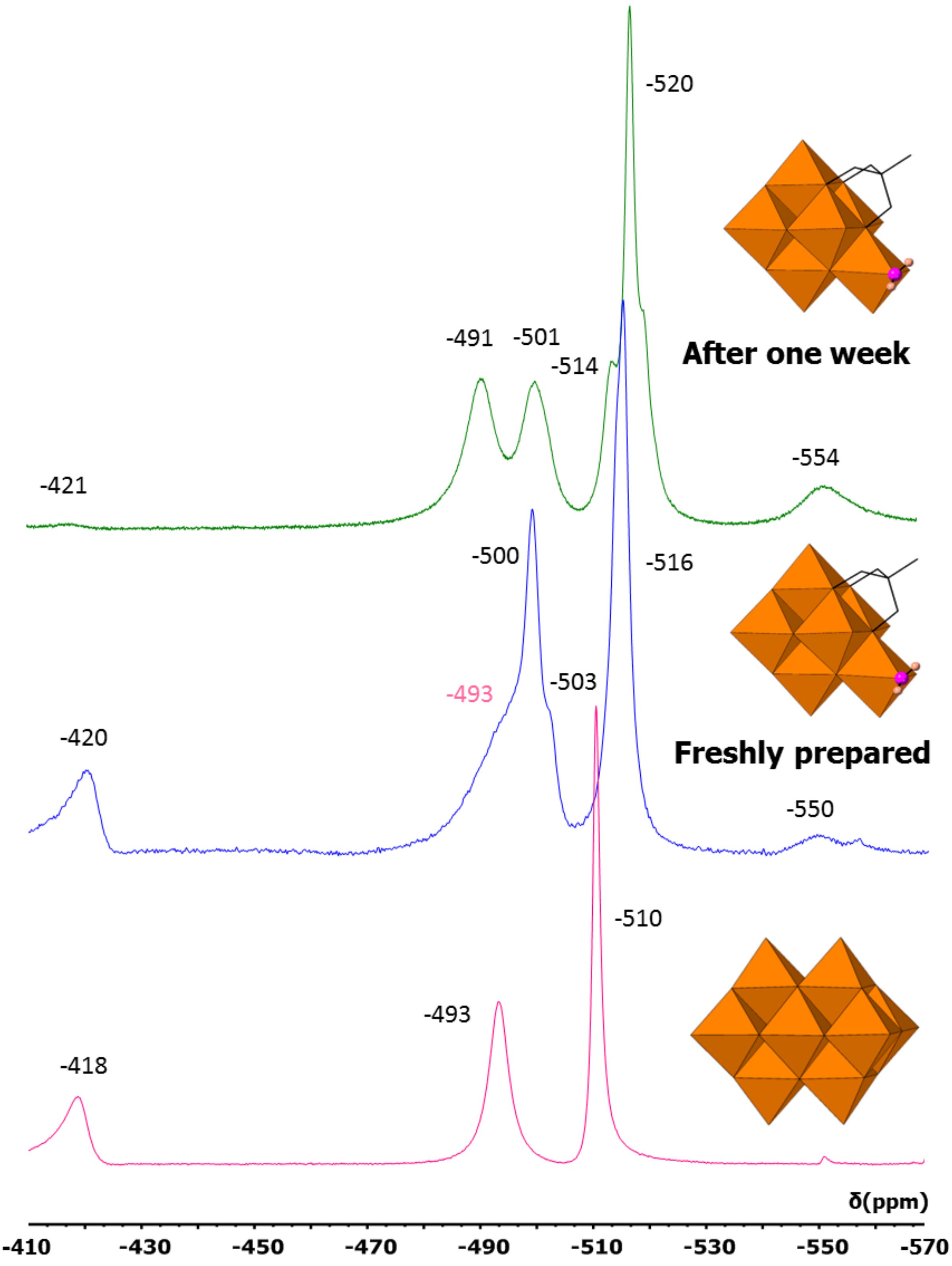

Figure S19. ^{51}V-NMR spectrum of a freshly prepared aqueous solution of **TMA-4** together with that recorded after 7 days and compared to that of the $Na_6[V_{10}O_{28}]\cdot 18H_2O$ provided as reference.
